# Diabetes mortality: trends and multi-country analysis of the Americas from 2000 to 2019

**DOI:** 10.1093/ije/dyad182

**Published:** 2024-01-10

**Authors:** Carmen Antini, Roberta Caixeta, Silvana Luciani, Anselm J M Hennis

**Affiliations:** Department of Noncommunicable Diseases and Mental Health, Pan American Health Organization, Washington, DC 20037, USA; Department of Noncommunicable Diseases and Mental Health, Pan American Health Organization, Washington, DC 20037, USA; Department of Noncommunicable Diseases and Mental Health, Pan American Health Organization, Washington, DC 20037, USA; Department of Noncommunicable Diseases and Mental Health, Pan American Health Organization, Washington, DC 20037, USA

**Keywords:** Mortality, trends, diabetes, diabetic kidney disease, chronic kidney disease, epidemiology

## Abstract

**Background:**

Diabetes has been increasing worldwide and is now among the 10 leading causes of death globally. Diabetic kidney disease (DKD), a complication of poorly managed diabetes, is related to high mortality risk. To better understand the situation in the Americas region, we evaluated diabetes and DKD mortality trends over the past 20 years.

**Methods:**

We analysed diabetes and DKD mortality for 33 countries in the Americas from 2000 to 2019. Data were extracted from the World Health Organization (WHO) Global Health Estimates and the World Population Prospects, 2019 Revision, estimating annual age-standardized mortality rates (ASMR) and gaps in the distribution of diabetes and DKD mortality by sex and country. Trend analyses were based on the annual average percentage of change (AAPC).

**Results:**

From 2000 to 2019, the overall mortality trend from diabetes in the Americas remained stable [AAPC: -0.2% (95% CI: -0.4%–0.0%]; however, it showed important differences by sex and by country over time. By contrast, DKD mortality increased 1.5% (1.3%–1.6%) per year, rising faster in men than women, with differences between countries. Central America, Mexico and the Latin Caribbean showed significant increases in mortality for both diseases, especially DKD. In contrast in North America, diabetes mortality decreased whereas DKD mortality increased.

**Conclusions:**

The increase in DKD mortality is evidence of poorly controlled diabetes in the region. The lack of programmes on prevention of complications, self-care management and gaps in quality health care may explain this trend and highlight the urgent need to build more robust health systems based on primary care, prioritizing diabetes prevention and control.

Key Messages

- Mortality trends in the Region of the Americas revealed wide variability in diabetes and diabetic kidney disease (DKD) mortality across countries and by sex, with stable diabetes mortality rates but a significant increase in mortality from DKD.
- Trends in mortality from diabetes and DKD and the sizeable excess mortality observed in some countries reflect the significance that diabetes has in mortality in the Americas, especially in Central America, Mexico and Latin Caribbean countries.
- Limited availability and access to quality health services for diabetes and DKD, including essential medicines, dialysis and renal transplant, may contribute to the high mortality due to diabetes and DKD, highlighting the urgent need for strengthened health systems based on primary care to improve diabetes diagnosis, treatment, prevention of diabetes-related complications and self-management support.

## Introduction

Diabetes represents a major public health challenge. Its global prevalence nearly doubled in only 30 years, showing a continuing upward trend.^1^ It is a multidimensional disease, driven by increasing obesity rates and characterized by high comorbidity, reduced quality of life, premature mortality and substantial economic and societal costs.^2–4^

People with diabetes are at increased risk of comorbid conditions such as cardiovascular diseases, chronic kidney disease (CKD), cancer and infectious diseases such as tuberculosis, influenza and COVID-19.^2–6^ Diabetes doubles the odds of CKD, and when combined with hypertension can increase this risk some 5-fold.^3^ Socioeconomic, cultural and political factors are determinants of CKD and diabetes and their inter-relationship, affecting countries' ability to respond to both diseases.^7^ To tackle this challenge, global and regional commitments and targets have been adopted by countries to halt the rise in diabetes and reduce premature mortality due to diabetes and other noncommunicable diseases (NCDs).^8–12^

The Global Health Estimates (GHE) of the World Health Organization (WHO) provide comprehensive and comparable time series data from 2000 onward on death and disability globally, by region and country, and by age, sex and cause. These data represent the WHO’s best estimates, calculated using standard categories, definitions and methods. In 2019, the WHO GHE reported nearly 1.5 million deaths directly caused by diabetes (excluding CKD), positioning it as the ninth leading cause of death globally.^13^ Diabetes-related CKD (DKD) mortality is reported separately in GHE, which is useful for measuring outcomes of complications from poorly controlled diabetes. However, it may also segment the magnitude of diabetes mortality. Few studies analyse mortality trends for both diabetes and DKD, especially in the Americas where diabetes and obesity are ranked among the world’s highest prevalence^14^ Given this limited available information and in support of the Pan American Health Organization Regional plan of action to prevent and control NCDs,^11^ this study aims to assess diabetes and DKD mortality and trends from 2000 to 2019 in the Region of the Americas by sex and year, at regional, subregional and country levels.

## Methods

This study focused on diabetes and DKD as the underlying cause of death for the 33 countries of the Americas included in the WHO GHE. The International Statistical Classification of Diseases and Related Health Problems 10th Revision (ICD-10) codes for diabetes were E10 (type 1 diabetes mellitus), E11 (type 2 diabetes mellitus), E12 (malnutrition-related diabetes mellitus), E13 (other specified diabetes mellitus) and E14 (unspecified diabetes mellitus), except for E10.2, E11.2, E12.2, E13.2, E14.2, which were codes applied to DKD. Data were not able to be processed separately for type 1 and type 2 diabetes because of how mortality data are recorded in the data source used.

We extracted the number of deaths from diabetes and DKD, with 95% confidence intervals (CI), by sex (male, female and both sexes combined), location (region, subregion and country), and year from the WHO GHE.^13^ The mid-year population used in the construction of rates was extracted from the estimates of the World Population Prospects, 2019 Revision.^15^ These data were used to estimate death rates (per 100 000 population) annually from 2000 to 2019 by sex and location.

To address the effect of different age distributions among populations and over time, we estimated age-standardized mortality rates (ASMR) using the direct standardization method, with the WHO World Standard Populations as reference.^16^ The gaps analysis in the geographical distribution of the diabetes and DKD mortality was based on the national excess mortality, defined as the ratio of the ASMR of a specific country and the regional ASMR. To characterize mortality differences by sex, we computed the male excess mortality, defined as the ratio of male ASMR relative to female ASMR, annually at regional, subregional and country levels.

Mortality trends from diabetes and DKD were analysed by applying the Joinpoint regression model, with the natural logarithm of each ASMR over time as the dependent variable, assuming homoscedasticity and an autocorrelation parameter. This model identifies inflection points for segments where the trend shows a significant change. We obtained the average annual percentage change (AAPC) as a summary measure of the changes over time from 2000 to 2019, with its corresponding 95% CI by sex and location. We considered an upward trend when both limits of the 95% CI were positive, a downward trend when both limits of the 95% CI were negative and a constant trend when the zero value was within the 95% CI of the AAPC. We used the Joinpoint Regression Program (version 4.9.0.0)^17^ for the time series analysis.

## Results

### Diabetes mellitus

**Figure 1.**
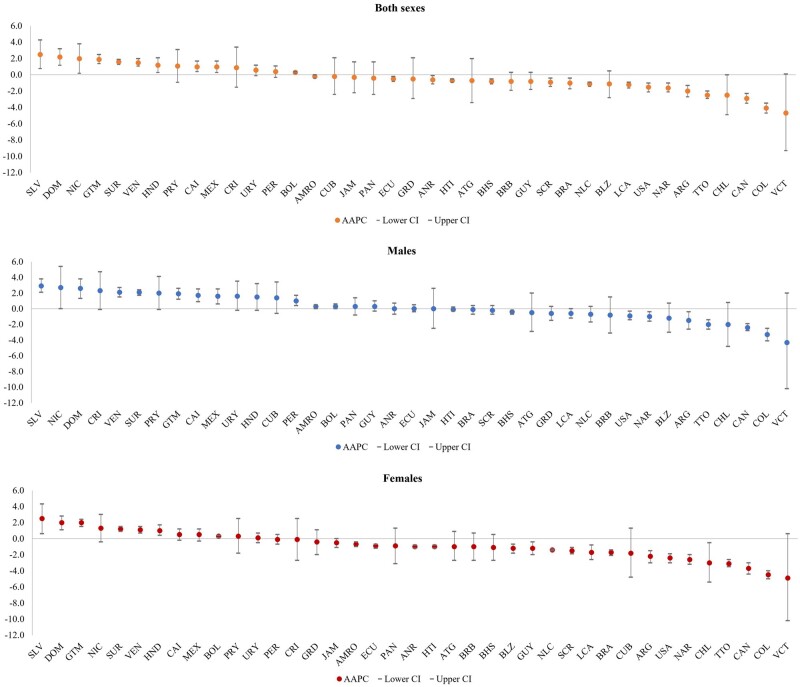
Average annual percent change for age-standardized mortality rate from diabetes mellitus by country and sex. Region of the Americas, 2000 to 2019. AMRO, Region of the Americas; ANR, Andean Area; BOL, Bolivia; Plurinational State of; COL, Colombia; ECU, Ecuador; PER, Peru; VEN, Venezuela, Bolivarian Republic of; CAI, Central America, Mexico and Latin Caribbean; CRI, Costa Rica; CUB, Cuba; DOM, Dominican Republic; GTM, Guatemala; HND, Honduras; HTI, Haiti; MEX, Mexico; NIC, Nicaragua; PAN, Panama; SLV, El Salvador; NLC, Non-Latin Caribbean; ATG, Antigua and Barbuda; BHS, Bahamas; BLZ, Belize; BRB, Barbados; GRD, Grenada; GUY, Guyana; JAM, Jamaica; LCA, Saint Lucia; SUR, Suriname; TTO, Trinidad and Tobago; VCT, Saint Vincent and the Grenadines; NAR, North America; CAN, Canada; USA, United States of America; SCR, Southern Cone and Brazil; ARG, Argentina; BRA, Brazil; CHL, Chile; PRY, Paraguay; URY, Uruguay.

In 2019, diabetes (excluding DKD) was reported as the underlying cause in 284 049 (95% CI: 231 879–342 108) deaths in the Americas, with the ASMR being highest in Guyana [82.6 per 100 000 population (58.4–112.0)] and lowest in Canada [7.2 per 100 000 population, (5.5–9.1)] (Table 1). In the 20-year period 2000 to 2019, diabetes was responsible for 4 509 047 deaths (53.2% in women). The regional ASMR decreased slightly from 21.9 per 100 000 population (19.6–24.1) in 2000 to 20.9 per 100 000 population (17.1–25.2) in 2019 with an AAPC of -0.2% [(−0.4%–0.0%), *P *=* *0.067], reflecting an overall stable trend in diabetes mortality. Among men, diabetes mortality increased 0.3% [(0.0%–0.5%), *P *=* *0.039] per year, whereas among women, it declined by −0.7% [(−1.0% – −0.4%), *P *<0.001] per year (Figure 1).

**Table 1.** Age-standardized mortality rates from diabetes mellitus (per 100 000 population) in the Region of the Americas, by country and sex for selected years

	Both sexes					Male					Female				
Location	2000	2005	2010	2015	2019	2000	2005	2010	2015	2019	2000	2005	2010	2015	2019
**Region of the Americas**	**21.9**	**22.4**	**21.1**	**20.7**	**20.9**	**22.2**	**23.4**	**22.4**	**22.6**	**23.1**	**21.7**	**21.5**	**19.9**	**19.1**	**18.9**
Andean Area	23.7	23.1	22.3	22.0	21.0	20.9	21.3	20.9	21.4	20.7	25.9	24.4	23.2	22.2	21.2
Bolivia, Plurinational State of	45.7	44.2	44.6	47.2	48.3	36.2	34.3	34.5	37.1	38.9	54.0	52.8	53.4	55.9	56.4
Colombia	19.9	17.8	12.7	10.7	9.1	15.9	15.4	10.3	9.6	8.6	23.1	19.6	14.5	11.7	9.5
Ecuador	29.6	34.3	32.7	29.2	26.6	26.7	32.5	31.4	28.8	26.7	31.9	35.7	33.7	29.3	26.4
Peru	12.4	11.7	15	14.2	13.3	10.8	10.4	14.5	13.8	13.0	13.7	12.7	15.3	14.4	13.3
Venezuela, Bolivarian Republic of	31.6	31.2	34.1	39.1	41.4	31.6	32.6	36.6	42.9	44.5	31.4	29.8	31.6	35.6	38.5
Central America, Mexico and Latin Caribbean	45.6	53.1	53.2	54.6	55.5	41.6	50.0	52.4	55.6	57.3	49.0	55.5	53.6	53.5	53.7
Costa Rica	11.2	11.2	9.2	10.2	14.9	9.8	9.6	8.4	10.3	14.4	12.5	12.4	9.9	9.9	15.1
Cuba	9.4	10.1	11.6	8.6	8.5	6.0	6.9	9.2	7.3	7.4	12.4	13.0	13.7	9.6	9.4
Dominican Republic	19.4	22.7	23.8	30.7	27.6	20.0	22.1	23.8	33.2	29.1	18.6	23.1	23.6	28.1	26.0
Guatemala	44.2	59.9	66.0	68.7	63.2	40.4	56.4	60.2	62.8	57.9	47.5	62.9	70.7	73.6	67.4
Honduras	13.9	15.0	15.1	19.8	17.5	11.1	11.8	11.9	19.2	15.6	16.2	17.8	17.7	20.3	19.2
Haiti	78.5	77.3	71.5	71.4	68.2	40.6	38.7	39.0	40.6	40.0	110.9	110.0	98.9	97.1	91.7
Mexico	59.5	69.2	68.2	69.9	71.8	57.7	69.1	71.3	74.8	77.9	60.8	69.1	65.3	65.4	66.4
Nicaragua	33.6	38.1	45.2	40.6	44.6	26.0	31.6	35.9	34.8	40.3	39.3	42.7	52.1	44.9	47.7
Panama	24.9	22.9	21.4	26.4	23.5	20.5	18.7	18.8	24.9	22.9	29.0	26.6	23.7	27.6	23.9
El Salvador	13.4	17.0	19.5	17.8	21.0	11.4	12.1	13.9	14.2	16.6	14.9	20.8	23.8	20.4	24.1
Non-Latin Caribbean	76.6	70.8	66.2	63.3	62.5	69.7	67.2	65.2	62.2	61.3	82.2	73.4	66.9	64.1	63.3
Antigua and Barbuda	58.7	64.6	52.5	47.2	51.1	50.5	59.1	48.5	44.4	44.8	65.6	69.6	56.0	49.0	56.3
Bahamas	45.7	44.1	42.4	39.2	40.3	42.7	42.2	41.7	39.7	41.3	47.4	45.1	42.7	38.5	39.1
Belize	67.9	64.0	59.7	52.6	53.8	62.0	53.2	49.5	46.6	48.7	73.4	74.0	70.3	59.2	59.1
Barbados	47.8	51.4	39.8	38.7	40.4	42.7	50.2	33.9	33.0	37.5	51.1	51.5	43.7	42.6	42.5
Grenada	70.1	62.2	82.4	65.1	65.2	71.6	67.4	79.8	63.8	68.2	69.3	57.3	85.9	64.2	62.5
Guyana	90.7	100.9	88.1	83.0	82.6	75.6	90.6	78.8	78.7	78.3	104.2	110.1	96.3	86.4	86.0
Jamaica	66.5	55.5	59.0	61.3	61.6	53.0	44.0	53.9	52.6	51.4	78.8	65.6	63.8	69.4	71.2
Saint Lucia	75.9	67.4	54.7	54.3	59.1	68.1	64	56.9	55.8	60.7	82.9	70.7	52.9	53.0	57.8
Suriname	39.8	41.2	43	45.5	53.7	41.7	45.2	47.3	51.2	61.4	38.0	37.8	39.2	40.9	47.6
Trinidad and Tobago	114	108.1	92.1	77.8	69.6	125.1	125.1	106.1	92.7	84.8	104.9	94.3	80.9	65.7	57.3
Saint Vincent and the Grenadines	103.1	93.2	57.8	56.7	46.0	75.4	78.7	48.5	50.9	37.3	126.6	105.0	67.4	62.3	54.2
North America	13.8	13.3	10.2	9.9	10.2	15.7	15.9	12.7	12.6	13.2	12.2	11.1	8.2	7.6	7.6
Canada	12.4	12.6	9.2	8.2	7.2	15.5	15.9	12.2	10.9	9.7	10.0	9.9	6.7	5.8	5.0
United States of America	13.9	13.4	10.4	10.1	10.6	15.8	15.9	12.8	12.8	13.6	12.4	11.3	8.3	7.8	7.9
Southern Cone and Brazil	25.6	23.7	24.5	22.2	21.3	24.7	24.0	25.1	23.7	23.4	26.0	23.4	23.9	21	19.4
Argentina	16.5	14.9	11.9	12.1	11.7	20.4	18.4	14.5	15.4	15.4	13.5	12.3	10.0	9.6	8.9
Brazil	30.8	27.9	30.0	25.9	24.6	27.7	26.6	29.4	26.5	26.3	33.0	28.7	30.2	25.2	23.1
Chile	13.8	14.8	13.3	13.7	11.0	15.4	17.0	15.6	16.3	13.1	12.6	13.2	11.5	11.7	9.3
Paraguay	33.4	45.5	40.5	42.8	41.8	24.0	36.2	34.0	38.5	38.1	41.5	53.3	46.0	46.1	44.8
Uruguay	10.7	10.1	10.3	11.8	12	11.8	12.9	12.9	14.8	14.8	9.7	8.2	8.7	9.8	9.9

Each cell represents an age-standardized mortality rate from diabetes mellitus (per 100 000 population), colored with a continuous color scale where the lighter color represents the lower rate and the darker color, the higher rate.

The greatest decline in diabetes mortality was observed in North America [Canada and the United States of America (USA)], with AAPCs of −2.6% [(−3.2% – −2.0%), *P *<0.001] in women and −1.0% [(−1.6% – −0.4%), *P *=* *0.001] in men. In contrast, Central America, Mexico and the Latin Caribbean were the subregions where increased diabetes mortality was evident [AAPC: 1.0% (0.4%–1.7%, *P *=* *0.001)], reaching 1.7% [(0.9%–2.5%), *P *<0.000] per year in men and 0.5% [(−0.2%–1.2%), *P *=* *0.148] (Figure 1).

Diabetes mortality increased faster in men, particularly in countries from Central America, Mexico and the Latin Caribbean, declined in six countries and was stable in 16 countries. Mortality in men demonstrated the largest increase in El Salvador, Nicaragua and the Dominican Republic, with AAPCs of 2.9% [(2.1%–3.8%), *P *<0.001], 2.7% [(0.0%–5.4%), *P *=* *0.048] and 2.6% [(1.3%–3.8%), *P *<0.001], respectively, whereas Colombia experienced the greatest decline [AAPC: −3.3% (−4.1% – −2.5%), *P *<0.001]. Among women, diabetes-related mortality increased in seven countries, declined in 12 countries and was stable in 14 countries. The greatest increase was in El Salvador, Dominican Republic and Guatemala, with AAPCs of 2.5% [(0.6%–4.3%), *P *=* *0.008], 2.0% [(1.1%–2.8%), *P *<0.001] and 2.0% [(1.5%–2.4%), *P *<0.001], respectively. Colombia documented the fastest decline [AAPC: −4.5% (−5.0% – −4.0%), *P *<0.001] (Figure 1; Supplementary Table S1, available as Supplementary data at *IJE* online).

National diabetes excess mortality showed wide variation within and between countries by sex and over time. The male ASMR in Central America, Mexico and Latin Caribbean was 1.87 times the regional rate in 2000, increasing to 2.49 times in 2019; excess mortality in women also increased, although to a lesser extent. In contrast, overall differences between subregional and regional rates in the Non-Latin Caribbean and North America decreasing in 2019 regarding 2000. Nationally, the highest excess mortality in 2019 was in Trinidad and Tobago for men and Haiti for women (Supplementary Table S2, available as Supplementary data at *IJE* online).

The male excess mortality increased in all subregions over the past two decades, with North America showing the largest change. In 2019, Canada and the USA documented an ASMR from diabetes in men almost twice the female rate. Other countries with the same dynamic were Argentina, Uruguay, Chile in the Southern Cone, Suriname, and Trinidad and Tobago in the Non-Latin Caribbean (Supplementary Table S3, available as Supplementary data at *IJE* online).

### Diabetic kidney disease

In the 20-year period under review, DKD was responsible for a total of 1 363 317 deaths (51.5% in women) in the Americas, with an ASMR increasing from 5.4 per 100 000 population (3.0–8.4) to 7.7 per 100 000 population (4.2–12.1) in men, and from 4.9 per 100 000 population (2.8–7.4) to 6.0 population (3.4–9.4) in women. The highest ASMR in 2019 was in Nicaragua in men [33.2 per 100 000 population (17.8–53.6)] and women [21.1 per 100 000 population (11.6–33.5)], followed by Mexico [21.1 per 100 000 population (12.1–32.1)] (Table 2).

**Table 2.** Age-standardized mortality rates from diabetic kidney disease (per 100 000 population) in the Region of the Americas, by country and sex for selected years

	Both sexes					Male					Female				
Location	2000	2005	2010	2015	2019	2000	2005	2010	2015	2019	2000	2005	2010	2015	2019
**Region of the Americas**	**5.1**	**6.1**	**6.6**	**6.9**	**6.8**	**5.4**	**6.5**	**7.2**	**7.7**	**7.7**	**4.9**	**5.7**	**6.1**	**6.2**	**6.0**
Andean Area	8.0	8.6	8.9	8.9	8.5	8.4	9.3	9.6	9.8	9.2	7.7	8.1	8.3	8.2	7.8
Bolivia, Plurinational State of	15.8	16.2	17.0	18.6	18.9	16.9	16.9	17.3	18.8	19.1	14.9	15.7	16.8	18.4	18.7
Colombia	5.2	4.9	4.5	4.8	3.9	5.4	5.4	4.8	5.4	4.5	5.1	4.5	4.3	4.3	3.4
Ecuador	11.3	15.6	15.2	13.4	12.1	11.8	16.7	16.6	14.4	13.0	10.9	14.6	13.9	12.5	11.5
Peru	6.6	6.5	7.6	6.7	6.5	5.9	6.1	7.4	6.4	6.1	7.1	6.9	7.8	6.9	6.8
Venezuela, Bolivarian Republic of	9.9	11.0	11.7	13.2	13.9	11.2	12.6	13.8	15.7	16.4	8.8	9.6	10.1	11.2	11.9
Central America, Mexico and Latin Caribbean	11.8	16.1	18.6	18.3	18.3	11.8	16.3	19.4	19.6	19.8	11.8	16.0	17.9	17.2	17.0
Costa Rica	7.6	9.0	8.7	8.0	11.4	8.8	10.0	9.7	9.3	12.8	6.6	8.2	7.9	6.9	10.3
Cuba	2.2	3.8	4.5	3.8	3.7	2.1	3.3	4.2	3.8	3.7	2.3	4.3	4.7	3.9	3.7
Dominican Republic	4.1	5.4	6.1	7.8	6.4	5.3	6.5	7.3	9.3	7.1	3.0	4.3	5.1	6.3	5.8
Guatemala	10.5	14.9	20.2	22.4	20.4	11.7	17.3	22.6	24.4	22.2	9.5	12.8	18.3	20.9	18.9
Honduras	11.8	14.4	15.2	20.3	17.9	10.1	11.7	12.3	19.5	15.6	13.2	16.8	17.7	21.1	19.9
Haiti	10.8	11.5	11.7	12.1	11.7	11.5	11.7	12.3	12.9	12.6	10.4	11.5	11.3	11.5	11.0
Mexico	15.2	20.9	23.7	22.6	22.7	14.9	20.9	24.6	24.1	24.6	15.5	20.9	22.9	21.4	21.1
Nicaragua	14.0	19.2	24.5	24.2	26.4	16.8	24.0	28.9	29.8	33.2	11.7	15.3	20.9	19.8	21.1
Panama	6.8	7.2	6.7	8.1	7.4	7.4	7.9	7.8	9.6	8.9	6.4	6.6	5.8	6.8	6.1
El Salvador	7.2	9.9	11.8	10.7	12.3	9.5	11.9	14.7	14.0	15.9	5.4	8.2	9.6	8.2	9.7
Non-Latin Caribbean	9.6	8.4	9.4	9.9	9.8	11.3	9.9	11.2	11.7	11.5	8.2	7.1	7.9	8.4	8.3
Antigua and Barbuda	8.7	10.4	9.7	10.0	10.4	9.8	11.5	10.8	11.8	11.2	7.8	9.6	8.9	8.6	9.8
Bahamas	7.8	9.5	10.3	10.2	10.4	9.3	10.8	11.6	11.7	12.0	6.7	8.5	9.3	8.9	9.1
Belize	12.6	12.0	12.6	12.7	12.7	14.3	12.3	12.6	13.1	13.4	10.9	11.7	12.6	12.2	12.0
Barbados	4.9	5.8	5.2	5.3	5.4	5.8	7.0	5.4	5.4	5.9	4.3	4.9	5.0	5.2	5.1
Grenada	11.4	11.1	15.8	15.2	15.3	16.4	15.3	20.2	18.3	19.2	7.9	7.9	13.0	12.4	12.3
Guyana	8.5	11.4	13.5	15.3	14.3	8.9	12.8	14.9	16.8	15.6	8.2	10.3	12.4	14.0	13.1
Jamaica	10.2	6.3	7.4	8.4	8.5	11.4	7.0	9.0	9.8	9.8	9.1	5.7	6.0	7.3	7.3
Saint Lucia	9.7	10.1	10.4	11.1	11.5	11.7	12.2	12.8	13.2	13.7	7.8	8.2	8.2	9.1	9.4
Suriname	12.4	13.9	14.2	15.7	18.2	15.1	16.6	17.3	19.3	22.4	10.2	11.7	11.7	13.0	15.2
Trinidad and Tobago	8.7	9.0	10.3	9.3	7.9	11.3	12.0	13.4	12.3	10.6	6.6	6.7	8.0	7.0	5.8
Saint Vincent and the Grenadines	9.4	10.4	7.7	8.5	6.9	9.7	11.2	7.9	8.8	6.6	9.4	10.0	7.8	8.3	7.2
North America	2.7	3.2	3.3	4.0	3.9	2.7	3.4	3.6	4.6	4.5	2.7	3.0	3.1	3.5	3.3
Canada	1.6	1.5	1.4	1.6	1.3	1.7	1.6	1.5	1.7	1.4	1.5	1.4	1.4	1.6	1.2
United States of America	2.8	3.4	3.5	4.3	4.2	2.8	3.6	3.9	4.9	4.9	2.8	3.2	3.3	3.7	3.5
Southern Cone and Brazil	5.6	5.4	5.3	5.1	4.8	6.7	6.4	6.4	6.2	6.0	4.9	4.6	4.6	4.3	4.0
Argentina	6.4	6.2	5.6	5.6	5.3	8.1	7.9	7.0	7.2	7.1	5.1	5.0	4.6	4.5	4.1
Brazil	5.5	5.0	5.2	4.8	4.6	6.3	5.9	6.2	5.7	5.6	4.8	4.3	4.5	4.1	3.8
Chile	4.9	5.8	5.3	5.6	4.4	5.6	6.4	6.1	6.5	5.3	4.4	5.3	4.8	4.9	3.8
Paraguay	6.6	8.8	9.8	12.0	11.2	6.4	9.0	10.4	13.3	12.1	6.9	8.6	9.4	10.9	10.3
Uruguay	2.9	3.3	3.2	3.8	3.9	3.9	4.8	4.5	5.3	5.2	2.1	2.4	2.4	3.0	3.0

Each cell represents an age-standardized mortality rate from diabetes mellitus (per 100 000 population), colored with a continuous color scale where the lighter color represents the lower rate and the darker color, the higher rate.

**Figure 2.**
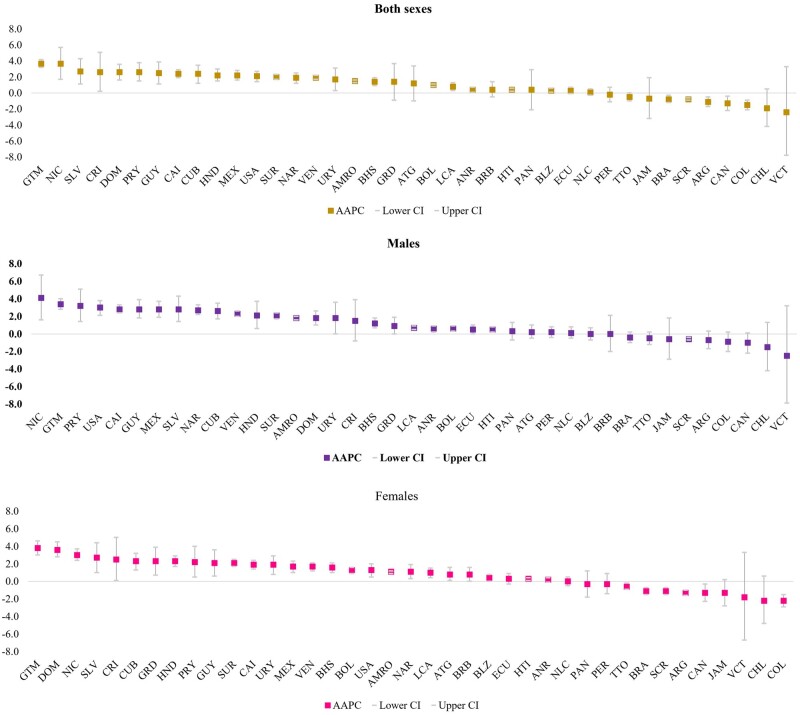
Average annual percent change for age-standardized mortality rate from diabetic kidney disease by country and sex Region of the Americas, 2000 to 2019. AMRO, Region of the Americas; ANR, Andean Area; BOL, Bolivia; Plurinational State of; COL, Colombia; ECU, Ecuador; PER, Peru; VEN, Venezuela, Bolivarian Republic of; CAI, Central America, Mexico and Latin Caribbean; CRI, Costa Rica; CUB, Cuba; DOM, Dominican Republic; GTM, Guatemala; HND, Honduras; HTI, Haiti; MEX, Mexico; NIC, Nicaragua; PAN, Panama; SLV, El Salvador; NLC, Non-Latin Caribbean; ATG, Antigua and Barbuda; BHS, Bahamas; BLZ, Belize; BRB, Barbados; GRD, Grenada; GUY, Guyana; JAM, Jamaica; LCA, Saint Lucia; SUR, Suriname; TTO, Trinidad and Tobago; VCT, Saint Vincent and the Grenadines; NAR, North America; CAN, Canada; USA, United States of America; SCR, Southern Cone and Brazil; ARG, Argentina; BRA, Brazil; CHL, Chile; PRY, Paraguay; URY, Uruguay.

Regional DKD mortality increased at an average (mean) annual rate of 1.5% [(1.3%–1.6%), *P *<0.001] from 2000 to 2019, rising faster in men [AAPC: 1.8% (1.6%–2.0%), *P *<0.001] than women [AAPC: 1.1% (0.9%–1.2%), *P *<0.001]. The largest increases were in Central America, Mexico and Latin Caribbean, and North America, with male AAPCs of 2.8% [(2.4%–3.3%), *P *<0.001] and 2.7% [(2.2%–3.3%), *P *<0.001], respectively. The upward trend for the female ASMR was slower, with AAPCs of 1.9% [(1.4%–2.4%), *P *<0.001] in Central America, Mexico and Latin Caribbean, and 1.1% ((0.3%–1.9%), *P *<0.005] in North America. The Southern Cone was the only subregion with a slow decline in men and women (Figure 2).

Nationally, the trend analysis revealed substantial increments in DKD mortality, with extreme AAPCs of 4.1% [(1.6%–6.7%), *P *<0.001; Nicaragua] in men and 3.8% [(2.9%–4.7%), *P *<0.001; Guatemala] in women. Interestingly, no country showed a decline in male mortality (Figure 2; Supplementary Table S1, available as Supplementary data at *IJE* online).

The ASMR from DKD showed persistent differences between countries over time. The comprehensive set of the ratio of national ASMR relative to regional ASMR from DKD by sex, country and year is in the Appendix (Supplementary Table S4, available as Supplementary data at *IJE* online). The male excess mortality from DKD increased in all subregions over time. In 2000, only Grenada had a male ASMR 50.0% higher than the female ASMR. Instead, in 2019, 10 (30.3%) of 33 countries documented a ratio of male-to-female mortality rate of over 1.5 (Supplementary Table S5, available as Supplementary data at *IJE* online).

## Discussion

At a time when the Americas region is in the phase of recovery from the COVID-19 pandemic, this multi-country analysis of the diabetes and DKD mortality is highly relevant and highlights the importance of rebuilding stronger health systems. Our analysis demonstrated three key outcomes. First, whereas mortality rates from diabetes have remained stable in the region over the past 20 years, the number of people dying from diabetes is increasing. Second, DKD mortality increased in most countries, likely an indication of poorly controlled diabetes and lack of programmes on the prevention of complications and self-care management.^4^^,^^18^^,^^19^ Third, there are profound differences in the mortality levels across countries for diabetes and DKD, signalling the inequities associated with diabetes care in the Americas region, at all levels of the health system.^4^^,^^18^^,^^20^

Consistent with previous reports,^21^^,^^22^ the highest ASMR from diabetes for the whole period was in the Non-Latin Caribbean; however, this subregion showed a downward trend, mainly driven by the dramatic decline observed in Trinidad and Tobago. Central America, Mexico and Latin Caribbean, the subregion with the second highest ASMR from diabetes, was the only subregion with an upward trend in mortality, particularly in men. This finding could be explained by the increases observed in the Dominican Republic, Nicaragua and El Salvador. Alarmingly, this subregion documented the fastest increase in DKD mortality, with upward trends in both men and women. This outcome is particularly worrying, given the co-existence of an epidemic of CKD unrelated to diabetes, hypertension or other known causes (non-traditional CKD) in several Central American countries since the 1970s.^23^

Our trend analysis revealed five different patterns in diabetes and DKD mortality: countries in which mortality from both diseases increased (nine countries); countries where diabetes mortality decreased but there were increases in DKD mortality (four countries); countries where mortality from both diseases remained stable over time (seven countries); and countries that demonstrated decreased ASMR in women for both diseases (five countries), whereas no country demonstrated reduced ASMRs in men. Of note, 12 (33.4%) of 33 countries showed no changes in male mortality from either diabetes or DKD.

It is noteworthy that in the USA, mortality from diabetes showed a downward trend over the past 20 years. According to the United States Renal Data System,^24^ the prevalence of DKD decreased from 41.5% in 2003–06 to 36.3% in 2015–18 among adults with diabetes, which contrasts with the rapid increase in DKD mortality evidenced in our study. Previous studies with similar results argue that this increase could be mainly due to DKD from type 2 diabetes and improvements in DKD screening.^2^^,^^7^ The increase in the age-standardized adult diabetes prevalence and the high DKD prevalence in people with type 2 diabetes at the time of diagnosis, reaching 25.0% after 10 years, support this argument.^25^^,^^26^ Unfortunately, the evidence shows that access to early detection and renal replacement therapy remains limited and variable across countries.^3^^,^^7^^,^^27^

The rapid increase in DKD mortality in most countries of the Americas is likely multifactorial, including an increasing prevalence of diabetes, population aging and impact of genetic and environmental risk factors; in conjunction with unhealthy lifestyles, all play a fundamental role.^2^^,^^4^^,^^7^ The pattern of DKD mortality is likely underpinned by factors such as underdiagnosis of diabetes, poor metabolic control, poor risk factor control and inequities in prevention, early diagnosis and management of DKD.^4^

Factors affecting availability and access to care may result in up to 70.0% of cases of diabetes being undiagnosed, depending on the country.^4^^,^^18^^,^^19^^,^^28^ In the Americas, this figure reaches 39.0%.^29^ Several factors must be considered when interpreting this high percentage; nevertheless, insufficient access to high-quality health care could play a pivotal role.^21^^,^^28^

Suboptimal control of hypertension as well as cholesterol associated with the development and progression of diabetes-related complications, especially, DKD.^3^^,^^4^ Use of statins for lipid management and renin-angiotensin system inhibitors for blood pressure reduce adverse cardio-renal events. Nevertheless in a recent survey, 30 of 35 (85.7%) countries in the Americas reported having statins generally available, (defined as the availability in 50.0% or more of the public sector pharmacies), and only 26 (74.3%) for angiotensin II receptors blockers.^20^

Intensive glycaemic control is known to affect outcomes in persons living with diabetes. An ancillary analysis of the 2019 Global Burden of Disease Study (GBD) reported that DKD accounted for 16.5% of all diabetes-related deaths in persons younger than 25 years, likely due extremely poor glycaemic control.^22^ Of particular concern, countries which reported decreases in insulin availability in the public sector pharmacies in 2021were among those noted to have the greatest increases in diabetes and DKD mortality.

Mortality is frequently used to assess the quality of health care because it is sensitive enough to reflect the performance of health systems.^18^ Reducing diabetes mortality requires a comprehensive approach that must consider comorbidities and combine efforts to ensure that patients have access to integrated, high-quality medical care at all levels of the health system.^18^^,^^30^ Primary care facilities are essential in this approach. The technical package HEARTS–D: Diagnosis and management of type 2 diabetes^31^: and the WHO Package of Essential Noncommunicable Disease Interventions in Primary Health Care (WHO-PEN)^32^ provide standardized guidance for the management and monitoring of diabetes, its related risk factors and other NCDs. They are valuable resources that, if fully implemented, can help countries improve health care quality for people with diabetes.

Our study has limitations, including methodological challenges related to the analysis of diabetes mortality. Our mortality data were limited to diabetes and DKD as the underlying cause of death, and this single-cause approach could mask deaths due to other diabetes-related complications, such as heart disease, stroke or even infectious diseases, classified under other ICD-10 codes. There is evidence that diabetes-related mortality can increase 3-fold if the analysis includes diabetes as an associated cause of death.^33^^,^^34^ Consequently, our results could be based on an underestimation of diabetes-related mortality.

Our findings contrast with previous studies documenting a decrease in mortality among people with diabetes. However, these reports show significant methodological differences from our study, mainly the population included in the denominator with which the mortality rates were estimated. Our study analyses diabetes as the underlying cause of death in the general population, and other studies analyse all causes of death among people with diabetes. Other critical methodological differences are the research time line and the data sources used.^35^^,^^36^ Magliano *et al.* analysed aggregate data on all-cause mortality in people with diabetes recollected by an international diabetes consortium across 16 high-income countries or jurisdictions from 1995 to 2016. Canada and the USA were the only countries from the Region of the Americas. In this study, deaths of people with diabetes were obtained by linkage to national death registries or national population registers, obtaining the standardized mortality ratio defined as the ratio of the observed number of deaths in people with diabetes to the expected number of deaths if mortality was similar to people without diabetes.^37^ The systematic review of Chen *et al.* focused on observational studies reporting all-cause mortality among people with diabetes, analysing mortality trends and differences between people with and without diabetes. Similar to the report of Magliano *et al*., the high-income countries of Canada and the USA were the two providing data from the Americas.^35^ Based on a cohort of Australians registered on the National Diabetes Services Scheme (NDSS) from 1997 to 2010, Harding *et al.*^36^ analysed secular trends in all-cause mortality in people with diabetes compared with the general population. In addition, this group evaluated the potential underestimation of cardiovascular death in people with diabetes. Our study therefore confirms findings of decreased DKD mortality in high-income countries in the Americas, and highlights the wide variation in mortality outcomes in a region known to have among the highest levels of socioeconomic disparities globally.

Another limitation is the different quality of vital registration data across countries.^22^^,^^34^^,^^38^ Our data were drawn from the WHO GHE, which has a comprehensive methodology to address this problem and achieve international comparability of mortality data, including revising vital registration data and adjusting country-cause-specific coding issues. Additionally, the GHE classifies countries according to the usability of their death data. Therefore, the interpretation of any result should consider this classification.^38^

Our findings revealed that regional diabetes mortality rates remained stable over the past two decades; however, DKD mortality increased in most countries. Given the significant impact that the COVID-19 pandemic has had on people with diabetes and DKD, it is likely continuing to rise.^39^ The high mortality rates for DKD reflect a multilevel challenge, where the limited availability and access to quality health services for diabetes and DKD, including dialysis and renal transplant, have a central role and may contribute to perpetuate this upward trend.^4^^,^^20^^,^^40^

The Region of the Americas faces an urgent need to improve health services for diabetes and DKD. The mortality trend pattern for both diseases revealed the importance of strengthening diagnosis, treatment and control rates for diabetes across countries that need urgent attention. It is necessary to scale up attention to controlling the key risk factors for NCDs (tobacco, harmful use of alcohol, unhealthy diet and physical inactivity).^41^^,^^42^

As the region recovers from the COVID-19 pandemic, it is critical that countries invest in improving the access to quality primary care services for prevention, screening, early diagnosis, treatment, prevention of complications and self-management support. The implementation of the Global Diabetes Compact^29^ in the Americas, together with the implementation of the 2022 WHO Executive Board commitment to strengthen national capacity for the prevention and control of diabetes,^9^ undoubtedly will help improve outcomes in diabetes.

## Conclusions

These trends and cross-country analysis showed that regional diabetes mortality remained stable in the last two decades. However, DKD mortality, one of the most frequent diabetes-related complications, increased rapidly in most countries. These findings evidenced the current focus on preventing and controlling diabetes appears insufficient. It needs to be strengthened by improving primary care services for diabetes. The Global Diabetes Compact and the 2022 WHO Executive Board's commitment will play a prominent role in improving outcomes in diabetes.

## Ethics approval

In this study, we analysed publicly available datasets of non-identifiable aggregated data, and the research was therefore exempt from formal ethics review.

## Supplementary Material

dyad182_Supplementary_Data

## Data Availability

The data underlying this study are available in the Global Health Estimates (GHE) of the World Health Organization (WHO) at [https://www.who.int/data/global-health-estimates].
