# Supplementary material for: Diabetes mortality: trends and multi-country analysis of the Americas from 2000 to 2019
Source: Int J Epidemiol. 2024 Jan 10;53(1):dyad182. doi: 10.1093/ije/dyad182 (PMC10859152; doi:10.1093/ije/dyad182)
Supplement: dyad182_Supplementary_Data [file dyad182_supplementary_data.docx]

**Supplementary material**

**Diabetes mortality: trends and multi-country analysis of the Americas from 2000 to 2019.**

**Table of Contents**

| **Table S1:** Average annual percent change for age-standardized mortality rate from diabetes mellitus and diabetic kidney disease (DKD), by country and sex. Region of the Americas, 2000 to 2019………………………………………………………………………………………………..…2 |
| --- |
| **Table S2:** Ratio of the national age-standardized mortality rate (ASMR) relative to the regional ASMR (per 100 000 population) for diabetes mellitus by sex, country, and year. Region of the Americas, 2000 to 2019………………………………………………………………………….4 |
| **Table S3:** Male excess mortality from diabetes mellitus by country and year. Region of the Americas, 2000 to 2019………………………………………………………………………………7 |
| **Table S4:** Ratio of the national age-standardized mortality rate (ASMR) relative to the regional ASMR (per 100 000 population) for diabetic kidney disease (DKD) by sex, country, and year. Region of the Americas, 2000 to 2019………………………………………………………....9 |
| **Table S5:** Male excess mortality from diabetic kidney disease (DKD) by country. Region of the Americas, 2000 to 2019…………………………………………………………………..….12 |
|  |

**Table S1.** Average annual percent change for age-standardized mortality rate from diabetes mellitus and diabetic kidney disease (DKD), by country and sex. Region of the Americas, 2000 to 2019.

|  | **Both sexes** | | |  | **Males** | | |  | | **Females** | |  |
| --- | --- | --- | --- | --- | --- | --- | --- | --- | --- | --- | --- | --- |
|  | **Diabetes** | **DKD** |  | **Diabetes** | | **DKD** |  | | **Diabetes** | | **DKD** | |
| **Region of the America** | **-0.2 (-0.4 – 0.0)** | **1.5* (1.3 – 1.6)** |  | **0.3* (0.0 – 0.5)** | | **1.8* (1.6 – 2.0)** |  | | **-0.7* (-1.0 – -0.4)** | | **1.1* (0.9 – 1.2)** | |
| **Andean Region** | **-0.6* (-1.1 – -0.1)** | **0.4* (0.1 – 0.7)** |  | **0.0 (-0.7 – 0.7)** | | **0.6* (0.2 – 0.9)** |  | | **-1.0* (-1.2 – -0.8)** | | **0.2 (-0.1 – 0.4)** | |
| Bolivia, Plurinational State of | 0.3* (0.2 – 0.4) | 1.0* (0.8 – 1.1) |  | 0.3* (0.0 – 0.6) | | 0.6* (0.3 – 0.9) |  | | 0.3* (0.1 – 0.4) | | 1.3* (0.9 – 1.6) | |
| Colombia | -4.1* (-4.7 – -3.5) | -1.5* (-2.1 – -0.9) |  | -3.3* (-4‘1 – -2.5) | | -0.9 (-2.0 – 0.2) |  | | -4.5* (-5.0 – -4.0) | | -2.2* (-2.9 – -1.5) | |
| Ecuador | -0.5* (-0.8 – -0.2) | 0.3 (-0.1 – 0.7) |  | 0.0 (-0.4 – 0‘5) | | 0.5 (0.0 – 1.0) |  | | -0.9* (-1.2 – -0‘7) | | 0.3 (-0.3 – 0.9) | |
| Peru | 0.4 (-0.3 – 1.1) | -0.2 (-1.1 – 0.7) |  | 1.0* (0.4 – 1.7) | | 0.2 (-0.4 – 0.8) |  | | -0.1 (-0.7 – 0.5) | | -0.3 (-1.4 – 0.9) | |
| Venezuela, Bolivarian Republic of | 1.5* (1.1 – 2.0) | 1.9* (1.7 – 2.2) |  | 2.1* (1.5 – 2.7) | | 2.3* (2.0 – 2.6) |  | | 1.1* (0.7 – 1.5) | | 1.7* (1.2 – 2.1) | |
|  |  |  |  |  | |  |  | |  | |  | |
| **Central America, Mexico and Latin Caribbean** | **1.0* (0.4 – 1.7)** | **2.4* (1.9 – 2.9)** |  | **1.7* (0.9 – 2.5)** | | **2.8* (2.4 – 3.3)** |  | | **0.5 (-0.2 – 1.2)** | | **1.9* (1.4 – 2.4)** | |
| Costa Rica | 0.9 (-1.5 – 3.4) | 2.6* (0.2 – 5.1) |  | 2.3 (-0.1 – 4.7) | | 1.5 (-0.8 – 3.9) |  | | -0.1 (-2.7 – 2.5) | | 2.5* (0.1 – 5.0) | |
| Cuba | -0.2 (-2.4 – 2.1) | 2.4* (1.2 – 3.5) |  | 1.4 (-0.6 – 3.4) | | 2.6* (1.7 – 3.5) |  | | -1.8 (-4.8 – 1.3) | | 2.3* (1.3 – 3.2) | |
| Dominican Republic | 2.2* (1.2 – 3.2) | 2.6* (1.6 – 3.6) |  | 2.6* (1.3 – 3.8) | | 1.8* (1.0 – 2.6) |  | | 2.0* (1.1 – 2.8) | | 3.6* (2.8 – 4.5) | |
| Guatemala | 1.9* (1.4 – 2.5) | 3.7* (3.2 – 4.2) |  | 1.9* (1.2 – 2.6) | | 3.4* (2.8 – 4.0) |  | | 2.0* (1.5 – 2.4) | | 3.8* (3.0 – 4.6) | |
| Honduras | 1.2* (0.3 – 2.1) | 2.2* (1.5 – 3.0) |  | 1.5 (-0.2 – 3.2) | | 2.1* (0.6 – 3‘7) |  | | 1.0* (0.4 – 1.7) | | 2.3* (1.7 – 2.9) | |
| Haiti | -0.7* (-0.9 – -0.5) | 0.4* (0.2 – 0.6) |  | -0.1 (-0.3 – 0.2) | | 0.5* (0.2 – 0.8) |  | | -1.0* (-1.2 – -0.8) | | 0.3* (0.1 – 0.4) | |
| Mexico | 1.0* (0.3 – 1.7) | 2.2* (1.6 – 2.8) |  | 1.6* (0.6 – 2.5) | | 2.8* (1.9 – 3.7) |  | | 0.5 (-0.3 – 1.2) | | 1.7* (1.0 – 2.3) | |
| Nicaragua | 2.0* (0.2 – 3.8) | 3.7* (1.7 – 5.7) |  | 2.7* (0.0 – 5.4) | | 4.1* (1.6 – 6.7) |  | | 1.3 (-0.4 – 3.0) | | 3.0* (2.4 – 3.7) | |
| Panama | -0.4 (-2.4 – 1.6) | 0.4 (-2.1 – 2.9) |  | 0.3 (-0.8 – 1.4) | | 0.3 (-0.7 – 1.3) |  | | -0.9 (-3.1 – 1.3) | | -0.3 (-1.8 – 1.2) | |
| El Salvador | 2.5* (0.8 – 4.3) | 2.7* (1.1 – 4.3) |  | 2.9* (2.1 – 3.8) | | 2.8* (1.4 – 4.3) |  | | 2.5* (0.6 – 4.3) | | 2.7* (1.0 – 4.4) | |
|  |  |  |  |  | |  |  | |  | |  | |
| **Non-Latin Caribbean** | **-1.1* (-1.4 – -0.9)** | **0.1 (-0.3 – 0.5)** |  | **-0.7 (-1.7 – 0.3)** | | **0.1 (-0.5 – 0.8)** |  | | **-1.4* (-1.5 – -1.3)** | | **0.0 (-0.5 – 0.5)** | |
| Antigua and Barbuda | -0.7 (-3.4 – 2.0) | 1.2 (-1.0 – 3.4) |  | -0.5 (-2.9 – 2.0) | | 0.2 (-0.5 – 1.0) |  | | -1.0 (-2.7 – 0.9) | | 0.8* (0.1 – 1.6) | |
| Bahamas | -0.8* (-1.1 – -0.5) | 1.4* (0.9 – 1.9) |  | -0.4* (-0.7 – -0.2) | | 1.2* (0.7 – 1.8) |  | | -1.1 (-2.7 – 0.5) | | 1.6* (1.0 – 2.1) | |
| Belize | -1.1 (-2.8 – 0.5) | 0.3* (0.1 – 0.6) |  | -1.2 (-3.0 – 0.7) | | 0.0 (-0.7 – 0.7) |  | | -1.2* (-1.8 – -0.7) | | 0.4 (0.0 – 0.8) | |
| Barbados | -0.8 (-1.9 – 0.3) | 0.4 (-0.5 – 1.4) |  | -0.8 (-3.1 – 1.5) | | 0.0 (-2.0 – 2.1) |  | | -1.0 (-2.7 – 0.7) | | 0.8* (0.0 – 1.6) | |
| Grenada | -0.5 (-2.9 – 2.1) | 1.4 (-0.9 – 3.7) |  | -0.6 (-1.5 – 0.3) | | 0.9 (0.0 – 1.9) |  | | -0.4 (-2.0 – 1.1) | | 2.3* (0.7 – 3.9) | |
| Guyana | -0.8 (-1.8 – 0.3) | 2.5* (1.1 – 3.9) |  | 0.3 (-0.3 – 1.0) | | 2.8* (1.8 – 3.9) |  | | -1.2* (-2.0 – -0.4) | | 2.1* (0.6 – 3.6) | |
| Jamaica | -0.3 (-2.2 – 1.6) | -0.7 (-3.2 – 1.9) |  | 0.0 (-2.5 – 2.6) | | -0.6 (-2.9 – 1.8) |  | | -0.5 (-1.1 – 0.0) | | -1.3 (-2.8 – 0.2) | |
| Saint Lucia | -1.2* (-1.6 – -0.9) | 0.8* (0.3 – 1.3) |  | -0.6 (-1.2 – 0.0) | | 0.7* (0.5 – 0.9) |  | | -1.7* (-2.6 – -0.8) | | 1.0* (0.4 – 1.5) | |
| Suriname | 1.6* (1.3 – 1.9) | 2.0* (1.7 – 2.3) |  | 2.1* (1.7 – 2.4) | | 2.1* (1.7 – 2.4) |  | | 1.2* (0.9 – 1.5) | | 2.1* (1.7 – 2.5) | |
| Trinidad and Tobago | -2.5* (-2.9 – -2.0) | -0.5* (-1.0 – 0.0) |  | -2.0* (-2.6 – -1.4) | | -0.5 (-1.2 – 0.2) |  | | -3.1* (-3.5 – -2.6) | | -0.6* (-0.9 – -0.2) | |
| Saint Vincent and the Grenadines | -4.7 (-9.3 – 0.1) | -2.4 (-7.8 – 3.3) |  | -4.3 (-10.2 – 2.0) | | -2.5 (-7.9 – 3.2) |  | | -4.9 (-10.2 – 0.6) | | -1.8 (-6.7 – 3.3) | |
|  |  |  |  |  | |  |  | |  | |  | |
| **North America** | **-1.6* (-2.1 – -1.0)** | **1.9* (1.2 – 2.5)** |  | **-1.0* (-1.6 – -0.4)** | | **2.7* (2.2 – 3.3)** |  | | **-2.6* (-3.2 – -2.0)** | | **1.1* (0.3 – 1.9)** | |
| Canada | -2.9* (-3.5 – -2‘3) | -1.3* (-2.2 – -0.4) |  | -2.4* (-2.8 – -1.9) | | -1.0 (-2.2 – 0.1) |  | | -3.7* (-4.4 – -3.0) | | -1.3* (-2.3 – -0.3) | |
| United States of America | -1.5* (-2.1 – -1.0) | 2.1* (1.4 – 2.7) |  | -0.9* (-1.4 – -0.3) | | 3.0* (2.1 – 3.8) |  | | -2.4* (-3.0 – -1.9) | | 1.3* (0.5 – 2.0) | |
|  |  |  |  |  | |  |  | |  | |  | |
| **Southern Cone** | **-0.9* (-1.4 – -0.4)** | **-0.8* (-0.9 – -0.6)** |  | **-0.2 (-0.7 – 0.4)** | | **-0.6* (-0.7 – -0.4)** |  | | **-1.5* (-1.9 – -1.1)** | | **-1.1* (-1.5 – -0.7)** | |
| Argentina | -2.0* (-2.7 – -1.3) | -1.1* (-1.7 – -0.5) |  | -1.5* (-2.6 – -0.4) | | -0.7 (-1.7 – 0.3) |  | | -2.2* (-3.0 – -1.5) | | -1.3* (-1.6 – -1.0) | |
| Brazil | -1.0* (-1.7 – -0.4) | -0.8* (-1.2 – -0.3) |  | -0.1 (-0.7 – 0.4) | | -0.4 (-1.0 – 0.2) |  | | -1.7* (-2.1 – -1.4) | | -1.1* (-1.5 – -0.7) | |
| Chile | -2.5* (-4.9 – 0.0) | -1.9 (-4.2 – 0.5) |  | -2.0 (-4.8 – 0.8) | | -1.5 (-4.2 – 1.3) |  | | -3.0* (-5.4 – -0.5) | | -2.2 (-4.8 – 0.6) | |
| Paraguay | 1.1 (-0.9 – 3.1) | 2.6* (1.5 – 3.8) |  | 2.0 (-0.1 – 4.1) | | 3.2* (1.4 – 5.1) |  | | 0.3 (-1.8 – 2.5) | | 2.2* (0.5 – 4.0) | |
| Uruguay | 0.6 (-0.1 – 1.2) | 1.7* (0.3 – 3.1) |  | 1.6 (-0.2 – 3.5) | | 1.8 (0.0 – 3.6) |  | | 0.1 (-0.5 – 0.7) | | 1.9* (0.8 – 2.9) | |

* The Average Annual Percetage Change is significantly different from zero at the alpha= 0·05 level.

**Table S2.** Ratio of the national age-standardized mortality rate (ASMR) relative to the regional ASMR (per 100 000 population) for diabetes mellitus by sex, country, and year. Region of the Americas, 2000 to 2019.

|  | **Male** | | | | | | | | | | | | | | | | | | | | |
| --- | --- | --- | --- | --- | --- | --- | --- | --- | --- | --- | --- | --- | --- | --- | --- | --- | --- | --- | --- | --- | --- |
| **Location Name** | 2000 | 2001 | 2002 | 2003 | 2004 | 2005 | 2006 | 2007 | 2008 | 2009 | 2010 | 2011 | 2012 | 2013 | 2014 | 2015 | 2016 | 2017 | 2018 | 2019 |  |
| **Andean Area** | **0.94** | **0.95** | **0.90** | **0.94** | **0.92** | **0.91** | **0.88** | **0.91** | **0.92** | **0.91** | **0.94** | **0.94** | **0.96** | **0.97** | **0.97** | **0.95** | **0.94** | **0.94** | **0.92** | **0.90** |  |
| Bolivia, Plurinational State of | 1.63 | 1.61 | 1.56 | 1.52 | 1.50 | 1.47 | 1.47 | 1.51 | 1.50 | 1.55 | 1.54 | 1.57 | 1.60 | 1.63 | 1.63 | 1.64 | 1.67 | 1.66 | 1.68 | 1.69 |  |
| Colombia | 0.72 | 0.73 | 0.69 | 0.71 | 0.68 | 0.66 | 0.63 | 0.60 | 0.57 | 0.46 | 0.46 | 0.44 | 0.45 | 0.42 | 0.40 | 0.42 | 0.39 | 0.39 | 0.38 | 0.37 |  |
| Ecuador | 1.21 | 1.22 | 1.30 | 1.29 | 1.35 | 1.39 | 1.40 | 1.40 | 1.43 | 1.40 | 1.41 | 1.43 | 1.45 | 1.41 | 1.31 | 1.28 | 1.26 | 1.25 | 1.20 | 1.16 |  |
| Peru | 0.49 | 0.47 | 0.46 | 0.46 | 0.46 | 0.45 | 0.46 | 0.48 | 0.50 | 0.60 | 0.65 | 0.65 | 0.66 | 0.67 | 0.64 | 0.61 | 0.59 | 0.59 | 0.58 | 0.57 |  |
| Venezuela, Bolivarian Republic of | 1.42 | 1.45 | 1.30 | 1.46 | 1.40 | 1.39 | 1.31 | 1.44 | 1.51 | 1.56 | 1.64 | 1.67 | 1.70 | 1.82 | 1.99 | 1.90 | 1.94 | 1.96 | 1.96 | 1.93 |  |
| **Central America, Mexico and Latin Caribbean** | **1.87** | **1.91** | **1.99** | **2.01** | **2.06** | **2.14** | **2.17** | **2.17** | **2.25** | **2.28** | **2.34** | **2.25** | **2.36** | **2.37** | **2.46** | **2.46** | **2.52** | **2.47** | **2.48** | **2.49** |  |
| Costa Rica | 0.44 | 0.43 | 0.35 | 0.52 | 0.51 | 0.41 | 0.38 | 0.30 | 0.35 | 0.33 | 0.38 | 0.32 | 0.28 | 0.33 | 0.32 | 0.46 | 0.51 | 0.57 | 0.59 | 0.62 |  |
| Cuba | 0.27 | 0.27 | 0.26 | 0.28 | 0.28 | 0.30 | 0.33 | 0.35 | 0.32 | 0.38 | 0.41 | 0.34 | 0.34 | 0.35 | 0.32 | 0.32 | 0.32 | 0.33 | 0.32 | 0.32 |  |
| Dominican Republic | 0.90 | 0.85 | 0.80 | 0.85 | 0.88 | 0.95 | 1.04 | 1.10 | 1.08 | 1.12 | 1.07 | 1.10 | 1.21 | 1.30 | 1.42 | 1.47 | 1.49 | 1.42 | 1.34 | 1.26 |  |
| El Salvador | 0.51 | 0.45 | 0.46 | 0.43 | 0.51 | 0.52 | 0.65 | 0.64 | 0.59 | 0.60 | 0.62 | 0.62 | 0.75 | 0.85 | 0.86 | 0.63 | 0.72 | 0.74 | 0.73 | 0.72 |  |
| Guatemala | 1.82 | 1.96 | 1.95 | 2.02 | 2.20 | 2.41 | 2.48 | 2.36 | 2.44 | 2.62 | 2.69 | 2.67 | 2.75 | 2.76 | 2.78 | 2.78 | 2.69 | 2.57 | 2.56 | 2.51 |  |
| Haiti | 1.83 | 1.78 | 1.71 | 1.67 | 1.66 | 1.66 | 1.70 | 1.76 | 1.76 | 1.81 | 1.75 | 1.81 | 1.84 | 1.84 | 1.82 | 1.80 | 1.76 | 1.75 | 1.75 | 1.74 |  |
| Honduras | 0.50 | 0.50 | 0.51 | 0.51 | 0.50 | 0.50 | 0.52 | 0.53 | 0.52 | 0.53 | 0.53 | 0.56 | 0.83 | 0.84 | 0.84 | 0.85 | 0.70 | 0.71 | 0.68 | 0.68 |  |
| Mexico | 2.60 | 2.64 | 2.80 | 2.80 | 2.86 | 2.95 | 2.98 | 2.96 | 3.09 | 3.10 | 3.19 | 3.03 | 3.17 | 3.17 | 3.31 | 3.31 | 3.42 | 3.34 | 3.36 | 3.38 |  |
| Nicaragua | 1.17 | 1.14 | 1.18 | 1.12 | 1.13 | 1.35 | 1.24 | 1.40 | 1.56 | 1.64 | 1.60 | 1.79 | 1.70 | 1.58 | 1.61 | 1.54 | 1.64 | 1.72 | 1.84 | 1.75 |  |
| Panama | 0.92 | 1.05 | 0.99 | 1.03 | 0.87 | 0.80 | 0.88 | 0.89 | 0.93 | 0.73 | 0.84 | 0.81 | 0.93 | 0.98 | 1.00 | 1.10 | 0.98 | 0.97 | 1.00 | 0.99 |  |
| **Non-Latin Caribbean** | **3.14** | **3.10** | **2.98** | **3.00** | **3.02** | **2.87** | **2.85** | **2.96** | **3.19** | **3.26** | **2.92** | **3.01** | **2.74** | **2.74** | **2.77** | **2.76** | **2.70** | **2.66** | **2.69** | **2.66** |  |
| Antigua and Barbuda | 2.28 | 2.44 | 2.67 | 2.85 | 2.57 | 2.53 | 1.99 | 2.34 | 2.41 | 2.54 | 2.17 | 2.09 | 1.84 | 1.85 | 1.78 | 1.97 | 1.90 | 2.12 | 1.95 | 1.94 |  |
| Bahamas | 1.92 | 1.98 | 1.76 | 1.73 | 1.90 | 1.80 | 1.91 | 1.80 | 1.82 | 1.87 | 1.86 | 1.85 | 1.86 | 1.72 | 1.72 | 1.76 | 1.76 | 1.73 | 1.78 | 1.79 |  |
| Barbados | 1.92 | 1.72 | 1.79 | 1.69 | 2.16 | 2.15 | 2.51 | 2.37 | 2.13 | 1.70 | 1.52 | 1.48 | 1.50 | 1.51 | 1.49 | 1.46 | 1.46 | 1.46 | 1.51 | 1.63 |  |
| Belize | 2.79 | 2.37 | 2.18 | 2.18 | 2.38 | 2.28 | 2.29 | 2.23 | 2.02 | 2.20 | 2.22 | 2.09 | 2.00 | 1.98 | 2.03 | 2.06 | 2.01 | 2.06 | 2.06 | 2.11 |  |
| Grenada | 3.23 | 3.13 | 3.32 | 3.11 | 3.37 | 2.88 | 2.92 | 2.74 | 2.92 | 3.37 | 3.57 | 3.41 | 2.41 | 2.41 | 2.71 | 2.83 | 3.00 | 2.98 | 3.11 | 2.96 |  |
| Guyana | 3.41 | 3.39 | 3.43 | 3.52 | 3.64 | 3.87 | 3.79 | 3.80 | 3.52 | 3.56 | 3.53 | 3.44 | 3.68 | 3.66 | 3.65 | 3.49 | 3.43 | 3.41 | 3.41 | 3.40 |  |
| Jamaica | 2.39 | 2.28 | 2.14 | 2.12 | 2.08 | 1.88 | 1.96 | 2.24 | 2.74 | 3.19 | 2.41 | 2.78 | 2.20 | 2.13 | 2.28 | 2.33 | 2.28 | 2.23 | 2.27 | 2.23 |  |
| Saint Lucia | 3.07 | 3.18 | 3.02 | 3.00 | 2.91 | 2.74 | 2.67 | 2.68 | 2.73 | 2.65 | 2.54 | 2.39 | 2.40 | 2.43 | 2.49 | 2.47 | 2.54 | 2.61 | 2.65 | 2.63 |  |
| Saint Vincent and the Grenadines | 3.40 | 3.27 | 3.78 | 3.93 | 4.04 | 3.37 | 2.69 | 1.78 | 1.50 | 1.46 | 2.17 | 2.88 | 3.42 | 3.47 | 2.90 | 2.25 | 1.63 | 1.39 | 1.65 | 1.62 |  |
| Suriname | 1.88 | 1.96 | 1.93 | 1.91 | 1.93 | 1.94 | 1.96 | 2.01 | 2.01 | 2.07 | 2.12 | 2.11 | 2.16 | 2.21 | 2.21 | 2.27 | 2.34 | 2.44 | 2.61 | 2.66 |  |
| Trinidad and Tobago | 5.64 | 5.74 | 5.46 | 5.66 | 5.52 | 5.35 | 5.01 | 4.98 | 5.25 | 4.61 | 4.74 | 4.31 | 4.25 | 4.35 | 4.24 | 4.11 | 4.00 | 3.88 | 3.78 | 3.68 |  |
| **North America** | **0.71** | **0.71** | **0.71** | **0.70** | **0.68** | **0.68** | **0.66** | **0.64** | **0.61** | **0.59** | **0.57** | **0.58** | **0.57** | **0.57** | **0.56** | **0.56** | **0.55** | **0.57** | **0.57** | **0.57** |  |
| Canada | 0.70 | 0.70 | 0.76 | 0.73 | 0.71 | 0.68 | 0.63 | 0.64 | 0.62 | 0.56 | 0.54 | 0.54 | 0.51 | 0.50 | 0.48 | 0.48 | 0.44 | 0.45 | 0.43 | 0.42 |  |
| United States of America | 0.71 | 0.71 | 0.71 | 0.70 | 0.68 | 0.68 | 0.66 | 0.64 | 0.61 | 0.60 | 0.57 | 0.59 | 0.58 | 0.58 | 0.57 | 0.57 | 0.56 | 0.58 | 0.58 | 0.59 |  |
| **Southern Cone** | **1.11** | **1.08** | **1.05** | **1.05** | **1.06** | **1.02** | **1.07** | **1.09** | **1.09** | **1.11** | **1.12** | **1.14** | **1.09** | **1.09** | **1.05** | **1.05** | **1.04** | **1.02** | **1.02** | **1.02** |  |
| Argentina | 0.92 | 0.91 | 0.84 | 0.88 | 0.84 | 0.79 | 0.73 | 0.76 | 0.67 | 0.66 | 0.65 | 0.65 | 0.65 | 0.65 | 0.62 | 0.68 | 0.70 | 0.63 | 0.66 | 0.67 |  |
| Brazil | 1.25 | 1.19 | 1.17 | 1.14 | 1.16 | 1.14 | 1.22 | 1.23 | 1.27 | 1.30 | 1.32 | 1.34 | 1.27 | 1.26 | 1.19 | 1.18 | 1.15 | 1.16 | 1.17 | 1.14 |  |
| Chile | 0.69 | 0.76 | 0.75 | 0.80 | 0.77 | 0.73 | 0.72 | 0.77 | 0.65 | 0.66 | 0.70 | 0.64 | 0.67 | 0.63 | 0.67 | 0.72 | 0.68 | 0.66 | 0.43 | 0.57 |  |
| Paraguay | 1.08 | 1.34 | 1.48 | 1.59 | 1.55 | 1.55 | 1.48 | 1.53 | 1.49 | 1.42 | 1.52 | 1.40 | 1.54 | 1.70 | 1.73 | 1.70 | 1.71 | 1.52 | 1.66 | 1.65 |  |
| Uruguay | 0.53 | 0.51 | 0.51 | 0.54 | 0.60 | 0.55 | 0.52 | 0.56 | 0.48 | 0.54 | 0.58 | 0.58 | 0.56 | 0.60 | 0.64 | 0.65 | 0.66 | 0.58 | 0.64 | 0.64 |  |
|  |  |  |  |  |  |  |  |  |  |  |  |  |  |  |  |  |  |  |  |  |  |
|  | **Females** | | | | | | | | | | | | | | | | | | | | |
|  | 2000 | 2001 | 2002 | 2003 | 2004 | 2005 | 2006 | 2007 | 2008 | 2009 | 2010 | 2011 | 2012 | 2013 | 2014 | 2015 | 2016 | 2017 | 2018 | 2019 |  |
| **Andean Area** | **1.20** | **1.19** | **1.17** | **1.18** | **1.16** | **1.14** | **1.13** | **1.13** | **1.14** | **1.15** | **1.16** | **1.15** | **1.19** | **1.18** | **1.20** | **1.16** | **1.15** | **1.15** | **1.13** | **1.12** |  |
| Bolivia, Plurinational State of | 2.49 | 2.47 | 2.45 | 2.41 | 2.43 | 2.45 | 2.49 | 2.59 | 2.60 | 2.68 | 2.68 | 2.77 | 2.83 | 2.89 | 2.90 | 2.92 | 2.95 | 2.96 | 2.98 | 2.98 |  |
| Colombia | 1.06 | 1.06 | 1.02 | 1.03 | 0.98 | 0.91 | 0.89 | 0.85 | 0.84 | 0.73 | 0.73 | 0.68 | 0.69 | 0.64 | 0.63 | 0.61 | 0.55 | 0.56 | 0.52 | 0.50 |  |
| Ecuador | 1.47 | 1.46 | 1.55 | 1.53 | 1.59 | 1.66 | 1.71 | 1.70 | 1.69 | 1.69 | 1.69 | 1.65 | 1.73 | 1.68 | 1.58 | 1.53 | 1.51 | 1.48 | 1.43 | 1.40 |  |
| Peru | 0.63 | 0.60 | 0.60 | 0.62 | 0.59 | 0.59 | 0.59 | 0.58 | 0.60 | 0.73 | 0.77 | 0.78 | 0.80 | 0.81 | 0.78 | 0.75 | 0.74 | 0.74 | 0.72 | 0.70 |  |
| Venezuela, Bolivarian Republic of | 1.45 | 1.45 | 1.42 | 1.43 | 1.42 | 1.38 | 1.36 | 1.43 | 1.48 | 1.55 | 1.59 | 1.59 | 1.68 | 1.71 | 1.95 | 1.86 | 1.92 | 1.98 | 1.99 | 2.04 |  |
| **Central America, Mexico and Latin Caribbean** | **2.26** | **2.31** | **2.36** | **2.45** | **2.50** | **2.58** | **2.58** | **2.53** | **2.63** | **2.64** | **2.69** | **2.60** | **2.66** | **2.69** | **2.78** | **2.80** | **2.84** | **2.81** | **2.83** | **2.84** |  |
| Costa Rica | 0.58 | 0.64 | 0.53 | 0.85 | 0.79 | 0.58 | 0.50 | 0.48 | 0.51 | 0.52 | 0.50 | 0.41 | 0.42 | 0.42 | 0.41 | 0.52 | 0.61 | 0.65 | 0.71 | 0.80 |  |
| Cuba | 0.57 | 0.56 | 0.46 | 0.51 | 0.58 | 0.60 | 0.59 | 0.59 | 0.61 | 0.66 | 0.69 | 0.58 | 0.55 | 0.54 | 0.52 | 0.50 | 0.48 | 0.50 | 0.49 | 0.49 |  |
| Dominican Republic | 0.86 | 0.86 | 0.94 | 0.98 | 1.07 | 1.08 | 1.13 | 1.16 | 1.17 | 1.21 | 1.18 | 1.21 | 1.27 | 1.35 | 1.42 | 1.47 | 1.46 | 1.44 | 1.40 | 1.38 |  |
| El Salvador | 0.69 | 0.79 | 0.69 | 0.64 | 0.75 | 0.97 | 1.03 | 1.04 | 0.99 | 1.12 | 1.19 | 1.18 | 1.39 | 1.59 | 1.51 | 1.06 | 1.27 | 1.30 | 1.29 | 1.27 |  |
| Guatemala | 2.19 | 2.26 | 2.38 | 2.50 | 2.66 | 2.93 | 3.08 | 3.16 | 3.13 | 3.46 | 3.55 | 3.61 | 3.68 | 3.78 | 3.81 | 3.85 | 3.78 | 3.66 | 3.61 | 3.56 |  |
| Haiti | 5.12 | 5.12 | 5.04 | 5.00 | 5.04 | 5.12 | 5.21 | 5.26 | 5.19 | 5.27 | 4.96 | 5.25 | 5.24 | 5.21 | 5.14 | 5.08 | 4.93 | 4.89 | 4.87 | 4.85 |  |
| Honduras | 0.75 | 0.75 | 0.75 | 0.76 | 0.81 | 0.83 | 0.85 | 0.87 | 0.87 | 0.88 | 0.89 | 0.93 | 1.02 | 1.04 | 1.05 | 1.06 | 1.02 | 1.03 | 1.02 | 1.02 |  |
| Mexico | 2.80 | 2.88 | 2.96 | 3.08 | 3.12 | 3.21 | 3.18 | 3.08 | 3.24 | 3.20 | 3.28 | 3.12 | 3.20 | 3.23 | 3.37 | 3.42 | 3.50 | 3.45 | 3.49 | 3.51 |  |
| Nicaragua | 1.81 | 1.77 | 1.90 | 1.87 | 2.02 | 1.99 | 2.12 | 2.12 | 2.49 | 2.43 | 2.61 | 2.59 | 2.46 | 2.33 | 2.39 | 2.35 | 2.42 | 2.59 | 2.58 | 2.52 |  |
| Panama | 1.34 | 1.32 | 1.56 | 1.41 | 1.39 | 1.24 | 1.38 | 1.19 | 1.24 | 1.09 | 1.19 | 1.29 | 1.41 | 1.38 | 1.47 | 1.44 | 1.22 | 1.29 | 1.28 | 1.26 |  |
| **Non-Latin Caribbean** | **3.79** | **3.80** | **3.60** | **3.55** | **3.52** | **3.41** | **3.41** | **3.48** | **3.41** | **3.37** | **3.36** | **3.32** | **3.34** | **3.28** | **3.35** | **3.35** | **3.29** | **3.31** | **3.32** | **3.35** |  |
| Antigua and Barbuda | 3.03 | 3.02 | 3.52 | 3.23 | 3.49 | 3.24 | 2.93 | 2.79 | 2.59 | 2.69 | 2.81 | 2.80 | 2.92 | 2.56 | 2.55 | 2.56 | 3.04 | 3.42 | 3.01 | 2.98 |  |
| Bahamas | 2.19 | 2.18 | 1.83 | 1.98 | 2.18 | 2.10 | 2.15 | 2.08 | 2.09 | 2.23 | 2.14 | 2.14 | 2.06 | 2.07 | 1.97 | 2.01 | 1.98 | 1.97 | 2.06 | 2.07 |  |
| Barbados | 2.36 | 2.45 | 2.31 | 2.63 | 2.49 | 2.39 | 2.46 | 2.74 | 2.71 | 2.42 | 2.19 | 2.15 | 2.26 | 2.23 | 2.23 | 2.23 | 2.22 | 2.24 | 2.25 | 2.24 |  |
| Belize | 3.39 | 3.27 | 3.27 | 3.29 | 3.50 | 3.44 | 3.68 | 3.59 | 3.31 | 3.51 | 3.53 | 3.48 | 3.44 | 3.38 | 3.32 | 3.09 | 2.99 | 3.04 | 3.05 | 3.13 |  |
| Grenada | 3.20 | 3.08 | 3.22 | 3.05 | 3.00 | 2.66 | 2.92 | 3.17 | 3.36 | 3.71 | 4.31 | 4.47 | 4.47 | 4.01 | 3.70 | 3.36 | 3.16 | 3.34 | 3.48 | 3.30 |  |
| Guyana | 4.81 | 5.53 | 5.24 | 5.18 | 4.99 | 5.12 | 5.14 | 5.09 | 4.76 | 4.85 | 4.83 | 5.01 | 4.85 | 4.59 | 4.37 | 4.51 | 4.48 | 4.53 | 4.54 | 4.55 |  |
| Jamaica | 3.64 | 3.61 | 3.36 | 3.17 | 3.15 | 3.05 | 3.07 | 3.22 | 3.15 | 3.26 | 3.20 | 3.26 | 3.24 | 3.22 | 3.56 | 3.63 | 3.58 | 3.60 | 3.65 | 3.76 |  |
| Saint Lucia | 3.82 | 3.70 | 3.57 | 3.44 | 3.41 | 3.29 | 3.29 | 2.96 | 2.82 | 2.77 | 2.65 | 2.65 | 2.64 | 2.64 | 2.73 | 2.77 | 2.93 | 3.01 | 3.05 | 3.06 |  |
| Saint Vincent and the Grenadines | 5.85 | 5.50 | 6.01 | 5.99 | 5.68 | 4.88 | 4.02 | 2.74 | 2.14 | 1.98 | 3.38 | 4.27 | 4.54 | 4.35 | 3.71 | 3.26 | 2.74 | 2.82 | 2.86 | 2.86 |  |
| Suriname | 1.76 | 1.83 | 1.83 | 1.77 | 1.79 | 1.76 | 1.79 | 1.84 | 1.87 | 1.95 | 1.96 | 2.00 | 2.04 | 2.08 | 2.09 | 2.14 | 2.21 | 2.32 | 2.46 | 2.52 |  |
| Trinidad and Tobago | 4.84 | 4.72 | 4.45 | 4.59 | 4.49 | 4.38 | 4.34 | 4.30 | 4.37 | 3.98 | 4.06 | 3.63 | 3.72 | 3.67 | 3.54 | 3.43 | 3.32 | 3.25 | 3.13 | 3.03 |  |
| **North America** | **0.56** | **0.56** | **0.56** | **0.54** | **0.52** | **0.52** | **0.49** | **0.47** | **0.46** | **0.43** | **0.41** | **0.42** | **0.41** | **0.41** | **0.40** | **0.40** | **0.39** | **0.40** | **0.40** | **0.40** |  |
| Canada | 0.46 | 0.49 | 0.50 | 0.49 | 0.47 | 0.46 | 0.41 | 0.41 | 0.41 | 0.37 | 0.34 | 0.35 | 0.33 | 0.32 | 0.32 | 0.30 | 0.29 | 0.28 | 0.27 | 0.27 |  |
| United States of America | 0.57 | 0.57 | 0.56 | 0.55 | 0.53 | 0.53 | 0.50 | 0.48 | 0.46 | 0.44 | 0.42 | 0.43 | 0.42 | 0.42 | 0.41 | 0.41 | 0.40 | 0.42 | 0.42 | 0.42 |  |
| **Southern Cone** | **1.20** | **1.16** | **1.14** | **1.11** | **1.12** | **1.09** | **1.14** | **1.19** | **1.16** | **1.19** | **1.20** | **1.21** | **1.16** | **1.15** | **1.10** | **1.10** | **1.08** | **1.06** | **1.05** | **1.03** |  |
| Argentina | 0.62 | 0.61 | 0.57 | 0.57 | 0.59 | 0.57 | 0.53 | 0.56 | 0.47 | 0.49 | 0.50 | 0.49 | 0.47 | 0.46 | 0.46 | 0.50 | 0.50 | 0.46 | 0.46 | 0.47 |  |
| Brazil | 1.52 | 1.45 | 1.44 | 1.39 | 1.38 | 1.33 | 1.43 | 1.49 | 1.48 | 1.51 | 1.51 | 1.53 | 1.46 | 1.42 | 1.35 | 1.32 | 1.29 | 1.27 | 1.27 | 1.22 |  |
| Chile | 0.58 | 0.66 | 0.61 | 0.62 | 0.65 | 0.61 | 0.58 | 0.59 | 0.51 | 0.52 | 0.58 | 0.53 | 0.57 | 0.55 | 0.58 | 0.61 | 0.62 | 0.57 | 0.37 | 0.49 |  |
| Paraguay | 1.91 | 1.94 | 2.14 | 2.27 | 2.33 | 2.48 | 2.35 | 2.50 | 2.44 | 2.23 | 2.31 | 2.27 | 2.36 | 2.60 | 2.53 | 2.41 | 2.39 | 2.23 | 2.38 | 2.37 |  |
| Uruguay | 0.45 | 0.46 | 0.41 | 0.45 | 0.43 | 0.38 | 0.43 | 0.43 | 0.41 | 0.45 | 0.43 | 0.46 | 0.45 | 0.47 | 0.49 | 0.51 | 0.53 | 0.48 | 0.51 | 0.53 |  |

**Table S3.** Male excess mortality from diabetes mellitus by country and year. Region of the Americas, 2000 to 2019.

|  | **Ratio of male ASMR^*^ relative to female ASMR from diabetes mellitus** | | | | | | | | | | | | | | | | | | | | |
| --- | --- | --- | --- | --- | --- | --- | --- | --- | --- | --- | --- | --- | --- | --- | --- | --- | --- | --- | --- | --- | --- |
| **Location Name** | 2000 | 2001 | 2002 | 2003 | 2004 | 2005 | 2006 | 2007 | 2008 | 2009 | 2010 | 2011 | 2012 | 2013 | 2014 | 2015 | 2016 | 2017 | 2018 | 2019 |  |
| **Region of the Americas** | **1.02** | **1.03** | **1.05** | **1.07** | **1.07** | **1.09** | **1.10** | **1.10** | **1.11** | **1.12** | **1.12** | **1.14** | **1.14** | **1.15** | **1.16** | **1.18** | **1.19** | **1.21** | **1.21** | **1.22** |  |
| **Andean Area** | **0.81** | **0.83** | **0.81** | **0.85** | **0.85** | **0.87** | **0.86** | **0.88** | **0.89** | **0.89** | **0.90** | **0.93** | **0.92** | **0.95** | **0.94** | **0.96** | **0.97** | **0.98** | **0.98** | **0.97** |  |
| Bolivia, Plurinational State of | 0.67 | 0.68 | 0.67 | 0.67 | 0.66 | 0.65 | 0.65 | 0.64 | 0.64 | 0.64 | 0.65 | 0.65 | 0.64 | 0.65 | 0.66 | 0.66 | 0.68 | 0.68 | 0.68 | 0.69 |  |
| Colombia | 0.69 | 0.71 | 0.71 | 0.74 | 0.75 | 0.78 | 0.77 | 0.77 | 0.76 | 0.71 | 0.71 | 0.74 | 0.75 | 0.76 | 0.74 | 0.82 | 0.84 | 0.85 | 0.88 | 0.91 |  |
| Ecuador | 0.84 | 0.87 | 0.89 | 0.90 | 0.91 | 0.91 | 0.90 | 0.91 | 0.94 | 0.92 | 0.93 | 0.98 | 0.95 | 0.97 | 0.97 | 0.98 | 0.99 | 1.02 | 1.01 | 1.01 |  |
| Peru | 0.79 | 0.81 | 0.81 | 0.79 | 0.83 | 0.82 | 0.86 | 0.91 | 0.91 | 0.92 | 0.95 | 0.95 | 0.94 | 0.95 | 0.95 | 0.96 | 0.96 | 0.97 | 0.97 | 0.98 |  |
| Venezuela, Bolivarian Republic of | 1.01 | 1.03 | 0.96 | 1.09 | 1.06 | 1.09 | 1.05 | 1.11 | 1.13 | 1.12 | 1.16 | 1.20 | 1.16 | 1.22 | 1.19 | 1.20 | 1.20 | 1.20 | 1.19 | 1.16 |  |
| **Central America, Mexico and Latin Caribbean** | **0.85** | **0.85** | **0.89** | **0.87** | **0.88** | **0.90** | **0.92** | **0.94** | **0.95** | **0.96** | **0.98** | **0.98** | **1.01** | **1.01** | **1.03** | **1.04** | **1.06** | **1.06** | **1.06** | **1.07** |  |
| Costa Rica | 0.78 | 0.69 | 0.69 | 0.66 | 0.69 | 0.77 | 0.83 | 0.69 | 0.78 | 0.71 | 0.85 | 0.90 | 0.76 | 0.92 | 0.91 | 1.04 | 1.00 | 1.04 | 1.00 | 0.96 |  |
| Cuba | 0.49 | 0.50 | 0.60 | 0.57 | 0.52 | 0.54 | 0.61 | 0.66 | 0.58 | 0.64 | 0.67 | 0.66 | 0.71 | 0.74 | 0.72 | 0.75 | 0.80 | 0.79 | 0.78 | 0.79 |  |
| Dominican Republic | 1.07 | 1.02 | 0.90 | 0.93 | 0.88 | 0.96 | 1.01 | 1.04 | 1.03 | 1.03 | 1.01 | 1.04 | 1.08 | 1.11 | 1.17 | 1.18 | 1.21 | 1.19 | 1.16 | 1.12 |  |
| El Salvador | 0.77 | 0.59 | 0.70 | 0.72 | 0.72 | 0.58 | 0.69 | 0.67 | 0.66 | 0.60 | 0.58 | 0.60 | 0.61 | 0.62 | 0.66 | 0.70 | 0.68 | 0.68 | 0.69 | 0.69 |  |
| Guatemala | 0.85 | 0.90 | 0.86 | 0.86 | 0.89 | 0.90 | 0.89 | 0.82 | 0.87 | 0.85 | 0.85 | 0.84 | 0.85 | 0.84 | 0.85 | 0.85 | 0.85 | 0.85 | 0.86 | 0.86 |  |
| Haiti | 0.37 | 0.36 | 0.36 | 0.36 | 0.35 | 0.35 | 0.36 | 0.37 | 0.38 | 0.38 | 0.39 | 0.39 | 0.40 | 0.41 | 0.41 | 0.42 | 0.42 | 0.43 | 0.44 | 0.44 |  |
| Honduras | 0.69 | 0.69 | 0.71 | 0.71 | 0.66 | 0.66 | 0.67 | 0.67 | 0.67 | 0.67 | 0.67 | 0.69 | 0.92 | 0.92 | 0.93 | 0.95 | 0.82 | 0.83 | 0.80 | 0.81 |  |
| Mexico | 0.95 | 0.95 | 0.99 | 0.97 | 0.99 | 1.00 | 1.03 | 1.06 | 1.06 | 1.08 | 1.09 | 1.11 | 1.13 | 1.13 | 1.14 | 1.14 | 1.16 | 1.17 | 1.17 | 1.17 |  |
| Nicaragua | 0.66 | 0.67 | 0.66 | 0.64 | 0.60 | 0.74 | 0.64 | 0.73 | 0.69 | 0.75 | 0.69 | 0.79 | 0.79 | 0.78 | 0.78 | 0.77 | 0.80 | 0.80 | 0.87 | 0.84 |  |
| Panama | 0.71 | 0.82 | 0.67 | 0.78 | 0.67 | 0.70 | 0.70 | 0.82 | 0.83 | 0.75 | 0.79 | 0.72 | 0.75 | 0.81 | 0.79 | 0.90 | 0.96 | 0.91 | 0.95 | 0.96 |  |
| **Non-Latin Caribbean** | **0.85** | **0.84** | **0.87** | **0.90** | **0.92** | **0.92** | **0.92** | **0.94** | **1.04** | **1.08** | **0.97** | **1.03** | **0.93** | **0.96** | **0.96** | **0.97** | **0.98** | **0.97** | **0.98** | **0.97** |  |
| Antigua and Barbuda | 0.77 | 0.84 | 0.80 | 0.94 | 0.79 | 0.85 | 0.75 | 0.92 | 1.03 | 1.06 | 0.87 | 0.85 | 0.72 | 0.83 | 0.81 | 0.91 | 0.74 | 0.75 | 0.78 | 0.80 |  |
| Bahamas | 0.90 | 0.94 | 1.01 | 0.93 | 0.94 | 0.94 | 0.98 | 0.95 | 0.96 | 0.94 | 0.98 | 0.98 | 1.03 | 0.95 | 1.01 | 1.03 | 1.06 | 1.06 | 1.05 | 1.06 |  |
| Barbados | 0.84 | 0.72 | 0.82 | 0.68 | 0.93 | 0.97 | 1.12 | 0.95 | 0.87 | 0.79 | 0.78 | 0.78 | 0.76 | 0.78 | 0.78 | 0.78 | 0.78 | 0.79 | 0.82 | 0.88 |  |
| Belize | 0.84 | 0.75 | 0.70 | 0.71 | 0.73 | 0.72 | 0.68 | 0.68 | 0.68 | 0.70 | 0.70 | 0.68 | 0.66 | 0.67 | 0.71 | 0.79 | 0.80 | 0.82 | 0.82 | 0.82 |  |
| Grenada | 1.03 | 1.05 | 1.09 | 1.08 | 1.20 | 1.18 | 1.10 | 0.95 | 0.96 | 1.01 | 0.93 | 0.87 | 0.61 | 0.69 | 0.85 | 0.99 | 1.13 | 1.07 | 1.08 | 1.09 |  |
| Guyana | 0.73 | 0.63 | 0.69 | 0.73 | 0.78 | 0.82 | 0.81 | 0.82 | 0.82 | 0.82 | 0.82 | 0.78 | 0.86 | 0.92 | 0.97 | 0.91 | 0.91 | 0.91 | 0.91 | 0.91 |  |
| Jamaica | 0.67 | 0.65 | 0.67 | 0.71 | 0.71 | 0.67 | 0.70 | 0.77 | 0.97 | 1.09 | 0.85 | 0.97 | 0.77 | 0.76 | 0.75 | 0.76 | 0.76 | 0.75 | 0.75 | 0.72 |  |
| Saint Lucia | 0.82 | 0.89 | 0.89 | 0.93 | 0.92 | 0.90 | 0.89 | 1.00 | 1.07 | 1.07 | 1.08 | 1.03 | 1.03 | 1.06 | 1.06 | 1.05 | 1.03 | 1.04 | 1.05 | 1.05 |  |
| Saint Vincent and the Grenadines | 0.60 | 0.61 | 0.66 | 0.70 | 0.76 | 0.75 | 0.74 | 0.71 | 0.78 | 0.82 | 0.72 | 0.77 | 0.86 | 0.92 | 0.91 | 0.82 | 0.71 | 0.60 | 0.70 | 0.69 |  |
| Suriname | 1.10 | 1.11 | 1.11 | 1.15 | 1.16 | 1.20 | 1.20 | 1.20 | 1.19 | 1.18 | 1.21 | 1.20 | 1.21 | 1.22 | 1.23 | 1.25 | 1.26 | 1.27 | 1.28 | 1.29 |  |
| Trinidad and Tobago | 1.19 | 1.26 | 1.29 | 1.31 | 1.32 | 1.33 | 1.27 | 1.27 | 1.33 | 1.29 | 1.31 | 1.35 | 1.30 | 1.36 | 1.39 | 1.41 | 1.43 | 1.44 | 1.46 | 1.48 |  |
| **North America** | **1.29** | **1.30** | **1.34** | **1.38** | **1.40** | **1.42** | **1.47** | **1.48** | **1.49** | **1.54** | **1.56** | **1.57** | **1.58** | **1.59** | **1.64** | **1.66** | **1.68** | **1.71** | **1.71** | **1.73** |  |
| Canada | 1.55 | 1.47 | 1.58 | 1.57 | 1.64 | 1.61 | 1.71 | 1.74 | 1.67 | 1.70 | 1.81 | 1.76 | 1.76 | 1.81 | 1.77 | 1.89 | 1.80 | 1.94 | 1.91 | 1.93 |  |
| United States of America | 1.27 | 1.29 | 1.32 | 1.36 | 1.38 | 1.41 | 1.45 | 1.46 | 1.47 | 1.53 | 1.53 | 1.55 | 1.56 | 1.57 | 1.63 | 1.64 | 1.67 | 1.69 | 1.70 | 1.71 |  |
| **Southern Cone** | **0.95** | **0.97** | **0.97** | **1.01** | **1.01** | **1.03** | **1.03** | **1.01** | **1.04** | **1.04** | **1.05** | **1.07** | **1.07** | **1.09** | **1.10** | **1.13** | **1.14** | **1.17** | **1.18** | **1.21** |  |
| Argentina | 1.51 | 1.56 | 1.56 | 1.63 | 1.51 | 1.50 | 1.53 | 1.48 | 1.57 | 1.50 | 1.45 | 1.51 | 1.59 | 1.61 | 1.57 | 1.61 | 1.69 | 1.65 | 1.72 | 1.73 |  |
| Brazil | 0.84 | 0.85 | 0.85 | 0.88 | 0.90 | 0.93 | 0.94 | 0.92 | 0.95 | 0.96 | 0.98 | 1.00 | 0.99 | 1.02 | 1.03 | 1.05 | 1.06 | 1.10 | 1.11 | 1.14 |  |
| Chile | 1.22 | 1.20 | 1.30 | 1.39 | 1.28 | 1.29 | 1.36 | 1.43 | 1.41 | 1.41 | 1.35 | 1.37 | 1.35 | 1.33 | 1.34 | 1.39 | 1.30 | 1.41 | 1.42 | 1.42 |  |
| Paraguay | 0.58 | 0.72 | 0.73 | 0.75 | 0.71 | 0.68 | 0.69 | 0.67 | 0.67 | 0.71 | 0.74 | 0.70 | 0.74 | 0.75 | 0.80 | 0.83 | 0.85 | 0.82 | 0.84 | 0.85 |  |
| Uruguay | 1.21 | 1.16 | 1.30 | 1.27 | 1.50 | 1.58 | 1.32 | 1.44 | 1.31 | 1.34 | 1.49 | 1.45 | 1.41 | 1.46 | 1.52 | 1.51 | 1.48 | 1.45 | 1.50 | 1.49 |  |

^*^Age-standardized mortality rate per 100 000 population.

**Table S4.** Ratio of the national age-standardized mortality rate (ASMR) relative to the regional ASMR (per 100 000 population) for diabetic kidney disease by sex, country, and year. Region of the Americas, 2000 to 2019.

|  | **Male** | | | | | | | | | | | | | | | | | | | | |
| --- | --- | --- | --- | --- | --- | --- | --- | --- | --- | --- | --- | --- | --- | --- | --- | --- | --- | --- | --- | --- | --- |
| **Location Name** | 2000 | 2001 | 2002 | 2003 | 2004 | 2005 | 2006 | 2007 | 2008 | 2009 | 2010 | 2011 | 2012 | 2013 | 2014 | 2015 | 2016 | 2017 | 2018 | 2019 |  |
| **Andean Area** | **1.55** | **1.53** | **1.46** | **1.49** | **1.46** | **1.43** | **1.37** | **1.37** | **1.37** | **1.34** | **1.34** | **1.32** | **1.31** | **1.30** | **1.29** | **1.26** | **1.24** | **1.24** | **1.22** | **1.20** |  |
| Bolivia, Plurinational State of | 3.11 | 3.03 | 2.90 | 2.76 | 2.68 | 2.58 | 2.53 | 2.50 | 2.44 | 2.46 | 2.40 | 2.42 | 2.41 | 2.42 | 2.47 | 2.44 | 2.43 | 2.44 | 2.47 | 2.48 |  |
| Colombia | 1.00 | 0.96 | 0.90 | 0.91 | 0.87 | 0.83 | 0.80 | 0.77 | 0.77 | 0.65 | 0.67 | 0.67 | 0.69 | 0.67 | 0.65 | 0.69 | 0.63 | 0.63 | 0.61 | 0.59 |  |
| Ecuador | 2.17 | 2.22 | 2.40 | 2.39 | 2.49 | 2.55 | 2.53 | 2.48 | 2.48 | 2.39 | 2.31 | 2.26 | 2.19 | 2.08 | 1.94 | 1.87 | 1.83 | 1.82 | 1.74 | 1.68 |  |
| Peru | 1.08 | 1.05 | 1.02 | 1.00 | 0.98 | 0.93 | 0.94 | 0.94 | 0.91 | 1.03 | 1.03 | 1.00 | 0.96 | 0.93 | 0.87 | 0.82 | 0.80 | 0.81 | 0.80 | 0.79 |  |
| Venezuela, Bolivarian Republic of | 2.07 | 2.07 | 1.84 | 2.04 | 1.96 | 1.93 | 1.76 | 1.86 | 1.88 | 1.88 | 1.92 | 1.89 | 1.87 | 1.96 | 2.13 | 2.03 | 2.09 | 2.14 | 2.15 | 2.13 |  |
| **Central America, Mexico and Latin Caribbean** | **2.16** | **2.23** | **2.34** | **2.36** | **2.42** | **2.50** | **2.58** | **2.61** | **2.70** | **2.70** | **2.69** | **2.55** | **2.60** | **2.55** | **2.56** | **2.53** | **2.58** | **2.55** | **2.57** | **2.57** |  |
| Costa Rica | 1.62 | 1.60 | 1.39 | 1.93 | 1.86 | 1.53 | 1.41 | 1.10 | 1.26 | 1.21 | 1.35 | 1.15 | 0.96 | 1.05 | 0.94 | 1.20 | 1.28 | 1.47 | 1.54 | 1.66 |  |
| Cuba | 0.39 | 0.46 | 0.47 | 0.51 | 0.49 | 0.50 | 0.53 | 0.54 | 0.48 | 0.56 | 0.58 | 0.49 | 0.49 | 0.51 | 0.49 | 0.49 | 0.49 | 0.49 | 0.48 | 0.48 |  |
| Dominican Republic | 0.97 | 0.92 | 0.88 | 0.93 | 0.93 | 0.99 | 1.04 | 1.06 | 1.04 | 1.07 | 1.01 | 1.04 | 1.10 | 1.14 | 1.21 | 1.21 | 1.17 | 1.08 | 1.00 | 0.92 |  |
| El Salvador | 1.74 | 1.57 | 1.67 | 1.57 | 1.82 | 1.82 | 2.18 | 2.11 | 1.97 | 2.01 | 2.04 | 2.00 | 2.36 | 2.61 | 2.55 | 1.81 | 2.06 | 2.12 | 2.10 | 2.07 |  |
| Guatemala | 2.15 | 2.25 | 2.19 | 2.23 | 2.45 | 2.65 | 2.71 | 2.63 | 2.79 | 3.01 | 3.15 | 3.13 | 3.20 | 3.21 | 3.20 | 3.15 | 3.04 | 2.95 | 2.93 | 2.88 |  |
| Haiti | 2.12 | 2.04 | 1.95 | 1.87 | 1.83 | 1.79 | 1.80 | 1.81 | 1.79 | 1.82 | 1.71 | 1.75 | 1.75 | 1.73 | 1.70 | 1.67 | 1.63 | 1.63 | 1.65 | 1.64 |  |
| Honduras | 1.86 | 1.84 | 1.87 | 1.84 | 1.79 | 1.79 | 1.81 | 1.79 | 1.73 | 1.73 | 1.71 | 1.78 | 2.53 | 2.53 | 2.52 | 2.52 | 2.11 | 2.18 | 2.05 | 2.03 |  |
| Mexico | 2.73 | 2.84 | 3.02 | 3.02 | 3.09 | 3.20 | 3.30 | 3.36 | 3.49 | 3.44 | 3.42 | 3.18 | 3.21 | 3.11 | 3.14 | 3.11 | 3.20 | 3.14 | 3.17 | 3.20 |  |
| Nicaragua | 3.08 | 3.09 | 3.33 | 3.05 | 3.16 | 3.68 | 3.32 | 3.64 | 4.00 | 4.18 | 4.01 | 4.49 | 4.26 | 3.95 | 4.00 | 3.86 | 4.09 | 4.34 | 4.61 | 4.31 |  |
| Panama | 1.36 | 1.55 | 1.49 | 1.47 | 1.31 | 1.21 | 1.28 | 1.22 | 1.28 | 0.96 | 1.09 | 1.01 | 1.10 | 1.10 | 1.11 | 1.24 | 1.12 | 1.10 | 1.16 | 1.16 |  |
| **Non-Latin Caribbean** | **2.07** | **1.97** | **1.77** | **1.68** | **1.63** | **1.51** | **1.55** | **1.55** | **1.66** | **1.70** | **1.56** | **1.63** | **1.52** | **1.50** | **1.54** | **1.52** | **1.49** | **1.48** | **1.51** | **1.50** |  |
| Antigua and Barbuda | 1.80 | 1.90 | 2.01 | 2.04 | 1.78 | 1.76 | 1.36 | 1.56 | 1.59 | 1.71 | 1.51 | 1.48 | 1.35 | 1.39 | 1.39 | 1.53 | 1.46 | 1.57 | 1.46 | 1.46 |  |
| Bahamas | 1.71 | 1.82 | 1.67 | 1.60 | 1.78 | 1.66 | 1.73 | 1.60 | 1.61 | 1.61 | 1.62 | 1.64 | 1.63 | 1.50 | 1.50 | 1.51 | 1.51 | 1.48 | 1.55 | 1.56 |  |
| Barbados | 1.06 | 0.97 | 1.02 | 0.92 | 1.14 | 1.08 | 1.22 | 1.11 | 1.01 | 0.83 | 0.76 | 0.72 | 0.75 | 0.74 | 0.72 | 0.69 | 0.69 | 0.69 | 0.72 | 0.76 |  |
| Belize | 2.64 | 2.25 | 2.05 | 1.99 | 2.08 | 1.88 | 1.85 | 1.76 | 1.59 | 1.73 | 1.76 | 1.67 | 1.62 | 1.58 | 1.65 | 1.70 | 1.66 | 1.69 | 1.69 | 1.74 |  |
| Grenada | 3.02 | 2.94 | 3.03 | 2.71 | 2.79 | 2.34 | 2.29 | 2.06 | 2.22 | 2.62 | 2.81 | 2.77 | 1.99 | 1.99 | 2.28 | 2.37 | 2.51 | 2.49 | 2.60 | 2.49 |  |
| Guyana | 1.64 | 1.69 | 1.75 | 1.78 | 1.83 | 1.96 | 1.98 | 2.03 | 1.93 | 2.00 | 2.08 | 2.14 | 2.39 | 2.37 | 2.37 | 2.18 | 2.09 | 2.06 | 2.04 | 2.02 |  |
| Jamaica | 2.10 | 1.86 | 1.52 | 1.37 | 1.24 | 1.07 | 1.19 | 1.27 | 1.50 | 1.65 | 1.25 | 1.42 | 1.15 | 1.13 | 1.24 | 1.27 | 1.25 | 1.25 | 1.28 | 1.27 |  |
| Saint Lucia | 2.15 | 2.19 | 2.08 | 2.01 | 1.94 | 1.87 | 1.88 | 1.84 | 1.88 | 1.81 | 1.78 | 1.66 | 1.72 | 1.73 | 1.77 | 1.71 | 1.71 | 1.76 | 1.79 | 1.78 |  |
| Saint Vincent and the Grenadines | 1.78 | 1.70 | 1.93 | 1.94 | 2.01 | 1.71 | 1.39 | 0.90 | 0.78 | 0.75 | 1.10 | 1.43 | 1.68 | 1.67 | 1.47 | 1.14 | 0.88 | 0.74 | 0.88 | 0.86 |  |
| Suriname | 2.78 | 2.90 | 2.84 | 2.72 | 2.63 | 2.55 | 2.54 | 2.50 | 2.42 | 2.45 | 2.41 | 2.39 | 2.45 | 2.50 | 2.49 | 2.50 | 2.56 | 2.68 | 2.86 | 2.91 |  |
| Trinidad and Tobago | 2.08 | 2.12 | 1.97 | 1.99 | 1.91 | 1.84 | 1.71 | 1.67 | 1.81 | 1.68 | 1.86 | 1.78 | 1.76 | 1.75 | 1.67 | 1.59 | 1.53 | 1.47 | 1.42 | 1.38 |  |
| **North America** | **0.50** | **0.51** | **0.51** | **0.51** | **0.51** | **0.52** | **0.51** | **0.50** | **0.50** | **0.50** | **0.51** | **0.55** | **0.56** | **0.58** | **0.58** | **0.59** | **0.58** | **0.59** | **0.59** | **0.59** |  |
| Canada | 0.32 | 0.30 | 0.30 | 0.28 | 0.26 | 0.24 | 0.22 | 0.22 | 0.21 | 0.20 | 0.20 | 0.22 | 0.21 | 0.22 | 0.22 | 0.22 | 0.20 | 0.20 | 0.19 | 0.18 |  |
| United States of America | 0.52 | 0.53 | 0.54 | 0.54 | 0.54 | 0.55 | 0.54 | 0.54 | 0.53 | 0.54 | 0.54 | 0.59 | 0.60 | 0.62 | 0.63 | 0.64 | 0.63 | 0.64 | 0.64 | 0.64 |  |
| **Southern Cone** | **1.22** | **1.16** | **1.10** | **1.07** | **1.05** | **0.98** | **0.96** | **0.95** | **0.90** | **0.89** | **0.89** | **0.87** | **0.83** | **0.82** | **0.79** | **0.80** | **0.79** | **0.78** | **0.77** | **0.77** |  |
| Argentina | 1.49 | 1.46 | 1.35 | 1.38 | 1.30 | 1.22 | 1.12 | 1.15 | 1.00 | 0.99 | 0.97 | 0.95 | 0.93 | 0.92 | 0.87 | 0.94 | 0.96 | 0.87 | 0.91 | 0.92 |  |
| Brazil | 1.16 | 1.08 | 1.02 | 0.97 | 0.96 | 0.90 | 0.91 | 0.88 | 0.87 | 0.87 | 0.86 | 0.86 | 0.79 | 0.78 | 0.74 | 0.74 | 0.72 | 0.73 | 0.75 | 0.73 |  |
| Chile | 1.03 | 1.10 | 1.05 | 1.09 | 1.05 | 0.98 | 0.94 | 0.98 | 0.82 | 0.82 | 0.86 | 0.75 | 0.78 | 0.74 | 0.79 | 0.84 | 0.80 | 0.80 | 0.52 | 0.68 |  |
| Paraguay | 1.17 | 1.37 | 1.42 | 1.44 | 1.39 | 1.38 | 1.32 | 1.37 | 1.34 | 1.32 | 1.44 | 1.38 | 1.55 | 1.71 | 1.77 | 1.72 | 1.69 | 1.48 | 1.60 | 1.57 |  |
| Uruguay | 0.72 | 0.67 | 0.71 | 0.74 | 0.80 | 0.74 | 0.66 | 0.68 | 0.55 | 0.60 | 0.63 | 0.63 | 0.60 | 0.64 | 0.68 | 0.68 | 0.69 | 0.62 | 0.69 | 0.68 |  |
|  |  |  |  |  |  |  |  |  |  |  |  |  |  |  |  |  |  |  |  |  |  |
|  | **Female** | | | | | | | | | | | | | | | | | | | | |
|  | 2000 | 2001 | 2002 | 2003 | 2004 | 2005 | 2006 | 2007 | 2008 | 2009 | 2010 | 2011 | 2012 | 2013 | 2014 | 2015 | 2016 | 2017 | 2018 | 2019 |  |
| **Andean Area** | **1.57** | **1.53** | **1.52** | **1.51** | **1.46** | **1.41** | **1.39** | **1.35** | **1.34** | **1.36** | **1.36** | **1.34** | **1.37** | **1.35** | **1.36** | **1.32** | **1.30** | **1.32** | **1.30** | **1.30** |  |
| Bolivia, Plurinational State of | 3.04 | 2.94 | 2.92 | 2.83 | 2.80 | 2.75 | 2.74 | 2.76 | 2.72 | 2.79 | 2.77 | 2.86 | 2.92 | 2.96 | 2.95 | 2.95 | 2.98 | 3.03 | 3.08 | 3.10 |  |
| Colombia | 1.03 | 0.99 | 0.94 | 0.93 | 0.87 | 0.79 | 0.77 | 0.74 | 0.75 | 0.68 | 0.71 | 0.69 | 0.72 | 0.70 | 0.70 | 0.69 | 0.62 | 0.63 | 0.58 | 0.56 |  |
| Ecuador | 2.23 | 2.28 | 2.49 | 2.48 | 2.52 | 2.57 | 2.60 | 2.50 | 2.43 | 2.38 | 2.30 | 2.22 | 2.29 | 2.20 | 2.07 | 2.01 | 1.99 | 1.96 | 1.94 | 1.90 |  |
| Peru | 1.46 | 1.35 | 1.33 | 1.34 | 1.25 | 1.21 | 1.16 | 1.09 | 1.09 | 1.27 | 1.28 | 1.26 | 1.25 | 1.23 | 1.17 | 1.11 | 1.09 | 1.11 | 1.12 | 1.13 |  |
| Venezuela, Bolivarian Republic of | 1.80 | 1.78 | 1.75 | 1.76 | 1.74 | 1.68 | 1.63 | 1.63 | 1.62 | 1.66 | 1.66 | 1.62 | 1.67 | 1.68 | 1.88 | 1.80 | 1.86 | 1.92 | 1.94 | 1.98 |  |
| **Central America, Mexico and Latin Caribbean** | **2.41** | **2.51** | **2.57** | **2.68** | **2.74** | **2.81** | **2.87** | **2.85** | **2.97** | **2.94** | **2.95** | **2.79** | **2.80** | **2.75** | **2.77** | **2.76** | **2.79** | **2.77** | **2.80** | **2.82** |  |
| Costa Rica | 1.36 | 1.48 | 1.25 | 1.88 | 1.86 | 1.43 | 1.30 | 1.23 | 1.31 | 1.34 | 1.30 | 1.07 | 1.10 | 1.05 | 0.98 | 1.11 | 1.25 | 1.37 | 1.50 | 1.70 |  |
| Cuba | 0.47 | 0.59 | 0.55 | 0.63 | 0.72 | 0.75 | 0.72 | 0.71 | 0.72 | 0.76 | 0.78 | 0.68 | 0.64 | 0.65 | 0.65 | 0.62 | 0.60 | 0.62 | 0.61 | 0.61 |  |
| Dominican Republic | 0.62 | 0.61 | 0.67 | 0.70 | 0.76 | 0.76 | 0.80 | 0.81 | 0.82 | 0.85 | 0.84 | 0.86 | 0.89 | 0.95 | 0.99 | 1.02 | 1.01 | 1.01 | 0.99 | 0.97 |  |
| El Salvador | 1.10 | 1.28 | 1.12 | 1.03 | 1.17 | 1.45 | 1.49 | 1.46 | 1.38 | 1.52 | 1.58 | 1.55 | 1.79 | 2.00 | 1.88 | 1.32 | 1.56 | 1.62 | 1.62 | 1.61 |  |
| Guatemala | 1.94 | 1.95 | 1.96 | 2.02 | 2.11 | 2.25 | 2.35 | 2.42 | 2.52 | 2.87 | 3.02 | 3.11 | 3.22 | 3.34 | 3.36 | 3.35 | 3.29 | 3.21 | 3.18 | 3.13 |  |
| Haiti | 2.12 | 2.08 | 2.05 | 2.01 | 2.00 | 2.01 | 2.02 | 2.01 | 1.95 | 1.98 | 1.87 | 1.93 | 1.90 | 1.89 | 1.87 | 1.84 | 1.80 | 1.80 | 1.82 | 1.82 |  |
| Honduras | 2.70 | 2.66 | 2.75 | 2.83 | 2.97 | 2.95 | 3.00 | 2.97 | 2.92 | 2.95 | 2.91 | 3.01 | 3.28 | 3.34 | 3.36 | 3.38 | 3.24 | 3.33 | 3.31 | 3.30 |  |
| Mexico | 3.16 | 3.29 | 3.38 | 3.52 | 3.56 | 3.67 | 3.74 | 3.72 | 3.90 | 3.79 | 3.78 | 3.52 | 3.50 | 3.41 | 3.43 | 3.44 | 3.48 | 3.42 | 3.47 | 3.49 |  |
| Nicaragua | 2.39 | 2.40 | 2.68 | 2.64 | 2.79 | 2.69 | 2.79 | 2.77 | 3.23 | 3.18 | 3.44 | 3.41 | 3.30 | 3.14 | 3.25 | 3.18 | 3.32 | 3.59 | 3.57 | 3.49 |  |
| Panama | 1.30 | 1.29 | 1.49 | 1.37 | 1.32 | 1.15 | 1.30 | 1.02 | 1.03 | 0.92 | 0.95 | 1.00 | 1.06 | 1.03 | 1.10 | 1.09 | 0.96 | 1.02 | 1.02 | 1.02 |  |
| **Non-Latin Caribbean** | **1.69** | **1.61** | **1.42** | **1.34** | **1.30** | **1.25** | **1.31** | **1.29** | **1.26** | **1.26** | **1.29** | **1.32** | **1.35** | **1.35** | **1.37** | **1.36** | **1.34** | **1.36** | **1.37** | **1.38** |  |
| Antigua and Barbuda | 1.60 | 1.57 | 1.84 | 1.70 | 1.80 | 1.69 | 1.51 | 1.43 | 1.34 | 1.44 | 1.47 | 1.48 | 1.62 | 1.44 | 1.43 | 1.38 | 1.66 | 1.86 | 1.63 | 1.62 |  |
| Bahamas | 1.37 | 1.43 | 1.32 | 1.37 | 1.56 | 1.49 | 1.56 | 1.48 | 1.47 | 1.55 | 1.53 | 1.52 | 1.47 | 1.49 | 1.43 | 1.44 | 1.40 | 1.39 | 1.49 | 1.50 |  |
| Barbados | 0.88 | 0.99 | 0.87 | 1.00 | 0.91 | 0.86 | 0.88 | 0.93 | 0.90 | 0.85 | 0.83 | 0.85 | 0.87 | 0.84 | 0.85 | 0.84 | 0.83 | 0.84 | 0.85 | 0.85 |  |
| Belize | 2.24 | 2.12 | 2.08 | 2.07 | 2.14 | 2.05 | 2.15 | 2.07 | 1.93 | 2.06 | 2.08 | 2.09 | 2.07 | 2.07 | 2.08 | 1.97 | 1.90 | 1.94 | 1.93 | 2.00 |  |
| Grenada | 1.62 | 1.54 | 1.60 | 1.54 | 1.51 | 1.38 | 1.45 | 1.53 | 1.56 | 1.73 | 2.14 | 2.24 | 2.33 | 2.18 | 2.13 | 1.99 | 1.89 | 2.09 | 2.21 | 2.05 |  |
| Guyana | 1.68 | 1.91 | 1.82 | 1.80 | 1.74 | 1.81 | 1.87 | 1.90 | 1.82 | 1.96 | 2.05 | 2.26 | 2.34 | 2.34 | 2.31 | 2.25 | 2.19 | 2.19 | 2.18 | 2.17 |  |
| Jamaica | 1.86 | 1.65 | 1.31 | 1.12 | 1.04 | 0.99 | 1.10 | 1.07 | 1.02 | 1.00 | 0.99 | 1.01 | 1.03 | 1.05 | 1.14 | 1.17 | 1.16 | 1.17 | 1.19 | 1.21 |  |
| Saint Lucia | 1.60 | 1.59 | 1.52 | 1.47 | 1.46 | 1.44 | 1.49 | 1.37 | 1.37 | 1.37 | 1.35 | 1.39 | 1.45 | 1.46 | 1.50 | 1.46 | 1.49 | 1.53 | 1.56 | 1.56 |  |
| Saint Vincent and the Grenadines | 1.92 | 1.80 | 2.05 | 2.06 | 1.98 | 1.75 | 1.50 | 1.01 | 0.80 | 0.70 | 1.28 | 1.61 | 1.71 | 1.64 | 1.46 | 1.34 | 1.12 | 1.12 | 1.18 | 1.19 |  |
| Suriname | 2.08 | 2.20 | 2.21 | 2.14 | 2.12 | 2.05 | 2.02 | 1.99 | 1.94 | 1.99 | 1.93 | 1.93 | 1.96 | 2.02 | 2.05 | 2.09 | 2.16 | 2.30 | 2.45 | 2.51 |  |
| Trinidad and Tobago | 1.35 | 1.31 | 1.23 | 1.25 | 1.23 | 1.18 | 1.20 | 1.20 | 1.25 | 1.21 | 1.31 | 1.24 | 1.30 | 1.24 | 1.18 | 1.13 | 1.08 | 1.05 | 1.00 | 0.96 |  |
| **North America** | **0.55** | **0.55** | **0.55** | **0.53** | **0.52** | **0.53** | **0.52** | **0.52** | **0.52** | **0.51** | **0.50** | **0.55** | **0.55** | **0.56** | **0.56** | **0.56** | **0.55** | **0.56** | **0.55** | **0.54** |  |
| Canada | 0.30 | 0.30 | 0.30 | 0.28 | 0.26 | 0.25 | 0.23 | 0.23 | 0.24 | 0.23 | 0.22 | 0.25 | 0.25 | 0.25 | 0.26 | 0.25 | 0.23 | 0.22 | 0.21 | 0.20 |  |
| United States of America | 0.58 | 0.58 | 0.57 | 0.56 | 0.55 | 0.56 | 0.56 | 0.56 | 0.55 | 0.54 | 0.54 | 0.59 | 0.59 | 0.60 | 0.59 | 0.60 | 0.59 | 0.60 | 0.59 | 0.59 |  |
| **Southern Cone** | **1.00** | **0.95** | **0.91** | **0.87** | **0.86** | **0.81** | **0.79** | **0.80** | **0.74** | **0.75** | **0.75** | **0.75** | **0.71** | **0.71** | **0.69** | **0.70** | **0.69** | **0.68** | **0.66** | **0.66** |  |
| Argentina | 1.05 | 1.01 | 0.94 | 0.92 | 0.93 | 0.88 | 0.80 | 0.85 | 0.72 | 0.73 | 0.75 | 0.72 | 0.68 | 0.68 | 0.68 | 0.72 | 0.71 | 0.67 | 0.67 | 0.67 |  |
| Brazil | 0.99 | 0.91 | 0.89 | 0.83 | 0.81 | 0.75 | 0.77 | 0.77 | 0.74 | 0.75 | 0.74 | 0.75 | 0.71 | 0.70 | 0.67 | 0.66 | 0.65 | 0.65 | 0.66 | 0.63 |  |
| Chile | 0.90 | 1.01 | 0.93 | 0.93 | 0.98 | 0.93 | 0.86 | 0.86 | 0.73 | 0.73 | 0.79 | 0.70 | 0.74 | 0.72 | 0.76 | 0.79 | 0.80 | 0.74 | 0.48 | 0.63 |  |
| Paraguay | 1.41 | 1.34 | 1.43 | 1.44 | 1.46 | 1.51 | 1.47 | 1.54 | 1.52 | 1.45 | 1.55 | 1.59 | 1.70 | 1.89 | 1.84 | 1.76 | 1.78 | 1.64 | 1.74 | 1.71 |  |
| Uruguay | 0.44 | 0.45 | 0.43 | 0.49 | 0.46 | 0.42 | 0.45 | 0.42 | 0.39 | 0.41 | 0.40 | 0.42 | 0.41 | 0.44 | 0.46 | 0.47 | 0.50 | 0.47 | 0.51 | 0.50 |  |

**Table S5.** Male excess mortality from diabetic kidney disease (DKD) by country. Region of the Americas, 2000 to 2019.

|  | **Ratio of male ASMR^*^ relative to female ASMR from DKD** | | | | | | | | | | | | | | | | | | | | |
| --- | --- | --- | --- | --- | --- | --- | --- | --- | --- | --- | --- | --- | --- | --- | --- | --- | --- | --- | --- | --- | --- |
| **Location Name** | 2000 | 2001 | 2002 | 2003 | 2004 | 2005 | 2006 | 2007 | 2008 | 2009 | 2010 | 2011 | 2012 | 2013 | 2014 | 2015 | 2016 | 2017 | 2018 | 2019 |  |
| **Region of the Americas** | **1.11** | **1.11** | **1.12** | **1.13** | **1.13** | **1.14** | **1.16** | **1.16** | **1.17** | **1.18** | **1.18** | **1.20** | **1.20** | **1.22** | **1.23** | **1.24** | **1.25** | **1.27** | **1.27** | **1.28** |  |
| **Andean Area** | **1.10** | **1.11** | **1.08** | **1.12** | **1.13** | **1.16** | **1.15** | **1.18** | **1.19** | **1.16** | **1.16** | **1.18** | **1.15** | **1.17** | **1.17** | **1.19** | **1.19** | **1.19** | **1.19** | **1.18** |  |
| Bolivia, Plurinational State of | 1.14 | 1.14 | 1.12 | 1.11 | 1.09 | 1.07 | 1.07 | 1.05 | 1.04 | 1.04 | 1.03 | 1.02 | 0.99 | 1.00 | 1.03 | 1.03 | 1.02 | 1.02 | 1.02 | 1.02 |  |
| Colombia | 1.07 | 1.07 | 1.07 | 1.11 | 1.14 | 1.20 | 1.21 | 1.21 | 1.20 | 1.12 | 1.12 | 1.16 | 1.16 | 1.17 | 1.14 | 1.24 | 1.27 | 1.27 | 1.32 | 1.35 |  |
| Ecuador | 1.08 | 1.08 | 1.09 | 1.09 | 1.12 | 1.14 | 1.13 | 1.15 | 1.19 | 1.18 | 1.19 | 1.22 | 1.15 | 1.15 | 1.15 | 1.15 | 1.15 | 1.17 | 1.14 | 1.13 |  |
| Peru | 0.83 | 0.86 | 0.86 | 0.85 | 0.89 | 0.89 | 0.93 | 0.99 | 0.97 | 0.95 | 0.96 | 0.95 | 0.93 | 0.93 | 0.92 | 0.92 | 0.92 | 0.92 | 0.91 | 0.90 |  |
| Venezuela, Bolivarian Republic of | 1.28 | 1.29 | 1.18 | 1.31 | 1.28 | 1.32 | 1.25 | 1.32 | 1.35 | 1.33 | 1.36 | 1.41 | 1.35 | 1.42 | 1.39 | 1.41 | 1.40 | 1.41 | 1.41 | 1.38 |  |
| **Central America, Mexico and Latin Caribbean** | **1.00** | **0.99** | **1.03** | **1.00** | **1.00** | **1.02** | **1.04** | **1.06** | **1.06** | **1.08** | **1.08** | **1.10** | **1.12** | **1.13** | **1.14** | **1.14** | **1.15** | **1.17** | **1.17** | **1.17** |  |
| Costa Rica | 1.33 | 1.20 | 1.24 | 1.16 | 1.14 | 1.22 | 1.26 | 1.03 | 1.13 | 1.07 | 1.23 | 1.30 | 1.05 | 1.23 | 1.18 | 1.34 | 1.27 | 1.36 | 1.31 | 1.24 |  |
| Cuba | 0.92 | 0.86 | 0.96 | 0.91 | 0.77 | 0.77 | 0.85 | 0.89 | 0.78 | 0.87 | 0.88 | 0.87 | 0.92 | 0.96 | 0.92 | 0.98 | 1.01 | 1.01 | 1.01 | 1.01 |  |
| Dominican Republic | 1.74 | 1.66 | 1.48 | 1.51 | 1.40 | 1.50 | 1.52 | 1.52 | 1.48 | 1.48 | 1.43 | 1.46 | 1.48 | 1.46 | 1.50 | 1.47 | 1.44 | 1.36 | 1.28 | 1.21 |  |
| El Salvador | 1.76 | 1.36 | 1.67 | 1.73 | 1.77 | 1.44 | 1.70 | 1.67 | 1.66 | 1.56 | 1.53 | 1.56 | 1.59 | 1.59 | 1.67 | 1.70 | 1.65 | 1.66 | 1.65 | 1.64 |  |
| Guatemala | 1.24 | 1.28 | 1.26 | 1.25 | 1.32 | 1.35 | 1.33 | 1.26 | 1.29 | 1.23 | 1.24 | 1.21 | 1.19 | 1.17 | 1.17 | 1.17 | 1.15 | 1.16 | 1.17 | 1.18 |  |
| Haiti | 1.11 | 1.09 | 1.07 | 1.05 | 1.04 | 1.02 | 1.03 | 1.05 | 1.07 | 1.08 | 1.09 | 1.09 | 1.11 | 1.12 | 1.12 | 1.12 | 1.13 | 1.15 | 1.15 | 1.15 |  |
| Honduras | 0.77 | 0.77 | 0.77 | 0.74 | 0.69 | 0.69 | 0.70 | 0.70 | 0.69 | 0.69 | 0.70 | 0.71 | 0.93 | 0.92 | 0.92 | 0.92 | 0.81 | 0.83 | 0.79 | 0.78 |  |
| Mexico | 0.96 | 0.96 | 1.00 | 0.97 | 0.99 | 1.00 | 1.02 | 1.05 | 1.04 | 1.07 | 1.07 | 1.09 | 1.10 | 1.11 | 1.13 | 1.13 | 1.15 | 1.16 | 1.16 | 1.17 |  |
| Nicaragua | 1.44 | 1.43 | 1.39 | 1.31 | 1.28 | 1.57 | 1.38 | 1.53 | 1.44 | 1.55 | 1.38 | 1.58 | 1.55 | 1.53 | 1.52 | 1.51 | 1.54 | 1.53 | 1.64 | 1.57 |  |
| Panama | 1.16 | 1.33 | 1.12 | 1.22 | 1.13 | 1.20 | 1.15 | 1.39 | 1.45 | 1.23 | 1.36 | 1.21 | 1.25 | 1.31 | 1.24 | 1.42 | 1.45 | 1.38 | 1.44 | 1.45 |  |
| **Non-Latin Caribbean** | **1.37** | **1.36** | **1.40** | **1.42** | **1.42** | **1.38** | **1.36** | **1.39** | **1.53** | **1.58** | **1.43** | **1.48** | **1.35** | **1.36** | **1.38** | **1.39** | **1.39** | **1.38** | **1.39** | **1.39** |  |
| Antigua and Barbuda | 1.25 | 1.35 | 1.23 | 1.36 | 1.12 | 1.20 | 1.05 | 1.27 | 1.39 | 1.40 | 1.22 | 1.20 | 1.01 | 1.17 | 1.19 | 1.38 | 1.10 | 1.07 | 1.14 | 1.15 |  |
| Bahamas | 1.39 | 1.41 | 1.43 | 1.33 | 1.30 | 1.27 | 1.28 | 1.25 | 1.28 | 1.23 | 1.25 | 1.30 | 1.34 | 1.23 | 1.29 | 1.31 | 1.35 | 1.35 | 1.32 | 1.32 |  |
| Barbados | 1.35 | 1.09 | 1.31 | 1.05 | 1.41 | 1.43 | 1.60 | 1.39 | 1.30 | 1.14 | 1.08 | 1.02 | 1.03 | 1.07 | 1.04 | 1.03 | 1.04 | 1.04 | 1.07 | 1.15 |  |
| Belize | 1.31 | 1.18 | 1.10 | 1.09 | 1.10 | 1.05 | 1.00 | 0.98 | 0.96 | 0.99 | 1.00 | 0.96 | 0.94 | 0.93 | 0.98 | 1.07 | 1.09 | 1.10 | 1.11 | 1.11 |  |
| Grenada | 2.07 | 2.13 | 2.13 | 2.00 | 2.10 | 1.94 | 1.83 | 1.56 | 1.65 | 1.78 | 1.56 | 1.48 | 1.03 | 1.11 | 1.32 | 1.48 | 1.66 | 1.51 | 1.49 | 1.55 |  |
| Guyana | 1.09 | 0.99 | 1.08 | 1.12 | 1.19 | 1.24 | 1.23 | 1.24 | 1.24 | 1.20 | 1.20 | 1.14 | 1.23 | 1.23 | 1.26 | 1.20 | 1.19 | 1.19 | 1.19 | 1.19 |  |
| Jamaica | 1.25 | 1.25 | 1.30 | 1.39 | 1.35 | 1.24 | 1.24 | 1.37 | 1.71 | 1.94 | 1.49 | 1.69 | 1.35 | 1.31 | 1.34 | 1.35 | 1.36 | 1.35 | 1.36 | 1.34 |  |
| Saint Lucia | 1.50 | 1.53 | 1.54 | 1.55 | 1.51 | 1.48 | 1.47 | 1.55 | 1.60 | 1.56 | 1.56 | 1.44 | 1.43 | 1.44 | 1.45 | 1.46 | 1.44 | 1.45 | 1.46 | 1.46 |  |
| Saint Vincent and the Grenadines | 1.03 | 1.05 | 1.06 | 1.06 | 1.15 | 1.12 | 1.07 | 1.03 | 1.13 | 1.25 | 1.02 | 1.07 | 1.18 | 1.24 | 1.24 | 1.06 | 0.97 | 0.84 | 0.95 | 0.93 |  |
| Suriname | 1.49 | 1.46 | 1.44 | 1.44 | 1.41 | 1.42 | 1.46 | 1.46 | 1.45 | 1.45 | 1.48 | 1.49 | 1.50 | 1.51 | 1.49 | 1.48 | 1.48 | 1.48 | 1.48 | 1.48 |  |
| Trinidad and Tobago | 1.72 | 1.80 | 1.79 | 1.80 | 1.77 | 1.79 | 1.66 | 1.62 | 1.69 | 1.64 | 1.69 | 1.71 | 1.63 | 1.71 | 1.74 | 1.75 | 1.77 | 1.78 | 1.81 | 1.82 |  |
| **North America** | **1.02** | **1.02** | **1.06** | **1.09** | **1.10** | **1.11** | **1.13** | **1.11** | **1.12** | **1.17** | **1.19** | **1.20** | **1.22** | **1.25** | **1.29** | **1.30** | **1.31** | **1.35** | **1.36** | **1.38** |  |
| Canada | 1.16 | 1.10 | 1.16 | 1.13 | 1.16 | 1.10 | 1.12 | 1.11 | 1.03 | 1.03 | 1.07 | 1.05 | 1.04 | 1.06 | 1.04 | 1.09 | 1.05 | 1.15 | 1.13 | 1.14 |  |
| United States of America | 1.01 | 1.02 | 1.05 | 1.09 | 1.10 | 1.11 | 1.13 | 1.12 | 1.13 | 1.18 | 1.20 | 1.21 | 1.23 | 1.26 | 1.30 | 1.31 | 1.33 | 1.36 | 1.37 | 1.40 |  |
| **Southern Cone** | **1.36** | **1.36** | **1.36** | **1.40** | **1.38** | **1.39** | **1.41** | **1.38** | **1.41** | **1.40** | **1.39** | **1.41** | **1.40** | **1.41** | **1.40** | **1.43** | **1.43** | **1.46** | **1.49** | **1.50** |  |
| Argentina | 1.59 | 1.62 | 1.61 | 1.70 | 1.58 | 1.58 | 1.62 | 1.57 | 1.63 | 1.59 | 1.53 | 1.58 | 1.65 | 1.65 | 1.57 | 1.61 | 1.69 | 1.66 | 1.73 | 1.74 |  |
| Brazil | 1.31 | 1.31 | 1.29 | 1.31 | 1.34 | 1.36 | 1.37 | 1.33 | 1.37 | 1.36 | 1.37 | 1.38 | 1.35 | 1.37 | 1.37 | 1.39 | 1.39 | 1.43 | 1.44 | 1.47 |  |
| Chile | 1.28 | 1.21 | 1.27 | 1.33 | 1.22 | 1.22 | 1.26 | 1.33 | 1.30 | 1.34 | 1.29 | 1.29 | 1.28 | 1.26 | 1.29 | 1.32 | 1.25 | 1.37 | 1.37 | 1.37 |  |
| Paraguay | 0.92 | 1.13 | 1.12 | 1.13 | 1.08 | 1.04 | 1.04 | 1.03 | 1.03 | 1.07 | 1.11 | 1.04 | 1.10 | 1.11 | 1.18 | 1.21 | 1.19 | 1.15 | 1.17 | 1.17 |  |
| Uruguay | 1.85 | 1.64 | 1.87 | 1.69 | 1.95 | 2.03 | 1.69 | 1.87 | 1.67 | 1.70 | 1.86 | 1.79 | 1.73 | 1.77 | 1.83 | 1.79 | 1.72 | 1.67 | 1.73 | 1.73 |  |

^*^Age-standardized mortality rate per 100 000 population.
